# Supplementary material for: Phasing analysis of lung cancer genomes using a long read sequencer
Source: Nat Commun. 2022 Jun 16;13:3464. doi: 10.1038/s41467-022-31133-6 (PMC9203510; doi:10.1038/s41467-022-31133-6)
Supplement: Supplementary file 1 — Supplementary Information [file 41467_2022_31133_MOESM1_ESM.pdf]

SUPPLEMENTARY INFORMATION

Phasing analysis of lung cancer genomes using a long read sequencer

Yoshitaka Sakamoto†, Shuhei Miyake†, Miho Oka†, Akinori Kanai, Yosuke Kawai, Satoi Nagasawa, Yuichi Shiraishi, Katsushi Tokunaga, Takashi Kohno, Masahide Seki, Yutaka Suzuki, Ayako Suzuki

†These authors contributed equally to this work.

Supplementary Methods (p. 2–p. 4)
Supplementary Figures S1–S17 (p. 5–pp. 40)
Supplementary Tables S1–S11 (pp. 41–pp. 56)
Supplementary References (pp. 57–pp. 58)

Supplementary Methods

Processing of short read WGS data for phasing analysis

Fastp is a tool for adapter trimming and filtering reads[1]. When filtering, the tool performs sliding window cutting of reads and checks their qualities. The tool also performs polyG tail trimming specific to Illumina NovaSeq data. We used this tool before mapping data to the human reference genome by default parameter. In particular, a base is qualified with 15 or more of the Phred quality score, and a read is filtered when over 40% of bases are unqualified. Moreover, a read is filtered when the number of N bases is greater than 5. Reads with less than 15 of read length are discarded. For sliding window cutting, the window size is four and the mean quality threshold of reads in the window is 20.

Reads after quality control were mapped to the human reference genome hg38 using BWA-MEM (version 0.7.17) by default parameter[2]. Then, the mapped reads were sorted, and PCR duplicates were marked using SAMtools (version 1.9)[3,4].

To detect germline variants, we used the GATK HaplotypeCaller (version 4.1.7.0)[5,6] with default parameters using the data of normal samples. In particular, GATK HaplotypeCaller assumes the ploidy as two. Before running the GATK HaplotypeCaller, we performed the base recalibration of input BAM files using known SNP or short indel sites from the 1000 Genomes Project phase 1[7], dbSNP[8], and Mills and 1000 Genomes Project gold standard indel data[9]. After running the GATK HaplotypeCaller, we performed the variant recalibration of the called SNPs using the 1000 Genomes Project phase 1, HapMap[10], dbSNP, and 1000 Genome Project Omni genotyping dataset. Then, we selected the variants passing the filters. The public data using the base and variant recalibrations were downloaded from the URL (https://console.cloud.google.com/storage/browser/genomics-public-data/resources/broad/hg38/v0); only the dbSNP data was from NCBI (https://www.ncbi.nlm.nih.gov/snp/).

Processing of long read WGS data

PromethION long read sequencing data were mapped to the human reference genome hg38 using Minimap2 (version 2.17) with the "-ax map-ont" option. Then, the mapped data were sorted and merged using SAMtools.

Haplotype phasing

Haplotype phasing was performed using the germline SNP data and mapped long read sequencing data by WhatsHap phase with the "--ignore-read-groups" option, meaning all

reads in the given BAM file come from the same sample (version 1.0)[11]. The WhatsHap phase command assumes the number of haplotypes as two (**Supplementary Fig. S6a**). When the number of haplotypes is ambiguous, haplotype phasing is difficult to resolve (**Supplementary Fig. S6b**). As cancer cells were heterogeneous because the mutations had been accumulating in tumor tissues during cancer evolution, an association between the mutations at a distance longer than reads by phasing analysis could not be guaranteed as actual phased events occurring in the same molecules. To actually "phase" multiple mutations at a distance longer than reads, which can be useful for analyzing cancer genomes, a single-cell analysis will be needed. The threshold of mapping quality is set to 20 by default, so low-quality reads, including reads with multiple hits (mapping quality: 0), were filtered out. In addition, WhatsHap uses only the primary alignment for haplotype phasing; the alignment information with supplementary flags is not used. This is because the supplementary reads were phased by other methods (see "*The detailed procedure of identification of somatic SVs and their haplotype information*"). WhatsHap haplotag command with the "--ignore-read-groups" option was used to add the haplotype tags to the reads in the long-read BAM files.

The detailed procedure of identification of somatic SVs and their haplotype information

Somatic SVs were detected from long read WGS data of tumor samples and their normal counterparts using Nanomonsv (version 0.1.2)[12] with default parameters. We filtered and classified the detected SVs using scripts provided on the Github page (https://github.com/friend1ws/nanomonsv). For the SV analysis, we used SVs in autosome. Supporting reads for each SV were extracted and annotated using the haplotype tag in the WhatsHap results. When mapped to the reference genome, the reads supporting SVs were generally split to ≥2 subreads, which were mapped to different regions of the reference genome (**Supplementary Fig. S17a**). In minimap2, one alignment of the subreads is assigned to "primary alignment" and the others are assigned to "supplementary alignment." WhatsHap only assigns haplotypes to subreads with primary alignment, so we manually counted the phased SNPs on the subreads with supplementary alignment supporting the SV using the SAMtools (version 1.7) mpileup function. Then, we extracted the subreads harboring ≥2 SNPs and a ratio of the number of SNPs for HP1 and HP2 ≥0.7 as a "phased subread." We defined those that were supported with ≥3 phased subreads and the ratio of the number of subreads for HP1 and HP2 ≥0.7 as "phased SVs" (**Supplementary Fig. S17b**).

The detailed procedure of methylation analysis

We used nanopolish software (version 0.13.2)[13] to call CpG methylation statuses from PromethION fast5 signal data and mapped BAM data using the "nanopolish call-methylation" command with default parameters. Reads with low mapping quality (<20) were filtered out. Using the haplotype-tagged PromethION reads from "WhatsHap haplotag," we classified the reads into two haplotypes and calculated the methylation frequencies in each haplotype category using the given script in nanopolish. DMRs between the haplotypes were detected by metilene (v.0.2-8) with default parameter. The threshold of adjusted p-value was set to 0.05. We called the DMRs in both tumor and normal specimens. Then, we defined "phase blocks with tumor-specific DMR bias" from the blocks satisfying the following conditions. (i) The number of DMRs that the methylation statuses and haplotypes were concordant being ≥3, e.g., high methylation status in HP1 (or HP2) and low methylation status in HP2 (or HP1) in each phase block; (ii) the ratio of (i) for total DMRs in each phase block being ≥0.7; (iii) the number of (i) per megabase (Mb) being ≥3. To accurately count (i), we excluded the DMRs in tumor samples that overlapped with over 20% of other DMRs in the matched-normal specimen. (**Supplementary Fig. S17c**).

The detailed procedure of transcriptome analysis

The obtained reads were aligned to the reference human genome hg38 using Minimap2 (version 2.2.17). To remove low-quality reads and reads of pseudogene mappings, aligned reads having the following conditions were discarded: (1) secondary or supplementary aligned flag; (2) mapping identity lower than 0.8; (3) unmapped length of reads within splice junctions longer than 10 bp; (4) exon length shorter than 25 bp; (5) overlapping the pseudogene region of GENCODE v27 [https://www.gencodegenes.org/].

Supplementary Figures

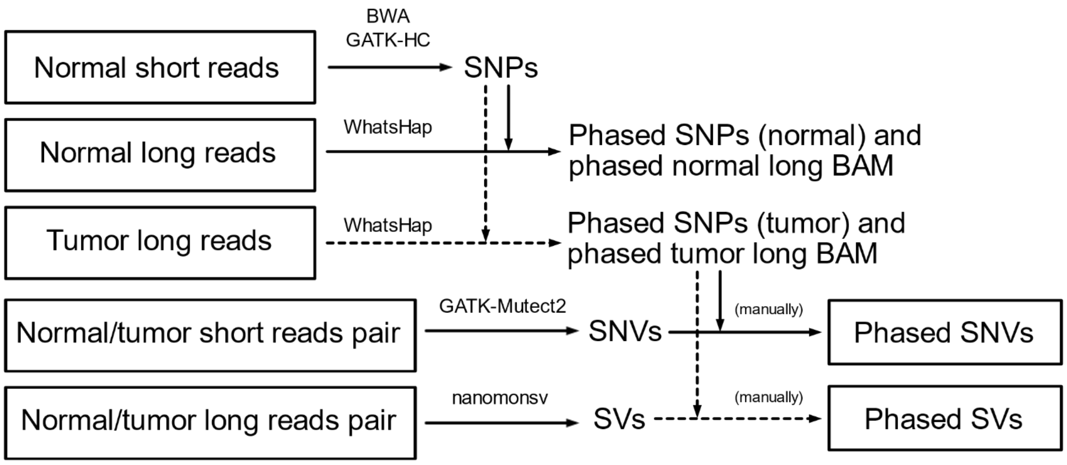

Supplementary Figure S1 A bioinformatics scheme of phasing analysis

The analytical scheme of the cancer phasing analysis described in this study. Employed bioinformatics pipelines and their associated tools for mapping, SNP detection, construction of phase blocks, and mutation assignments to haplotypes are shown in the workflow.

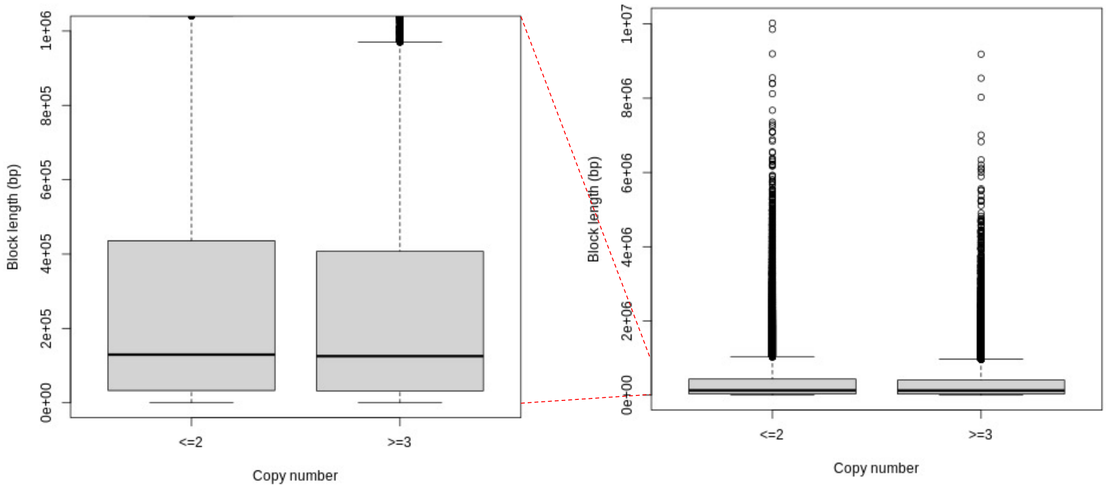

Supplementary Figure S2 Relationship of copy number (CN) and block length

Boxplot of block length by CN. Phase blocks in the regions with ≤2 of CN had comparable block length with those in the regions with ≥3 of CN. The ends of the box plots indicate lower and upper quartiles; center line, median; whiskers, maximum and minimum values except with outliers, respectively.

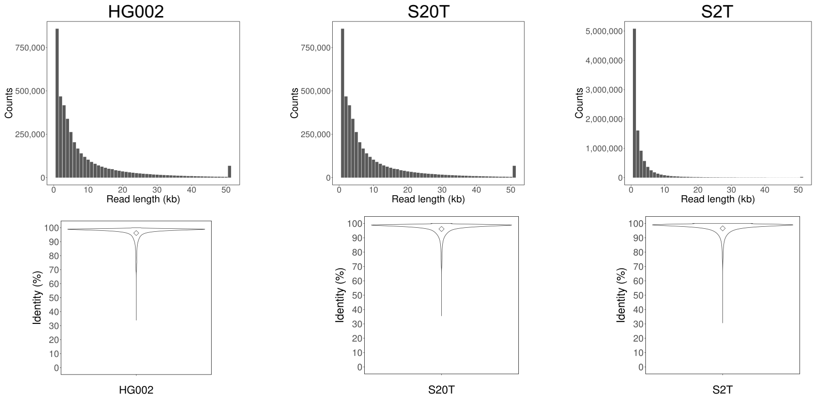
HG002
S20T
S2T
Counts
Read length (kb)
Identity (%)
HG002
S20T
S2T

b

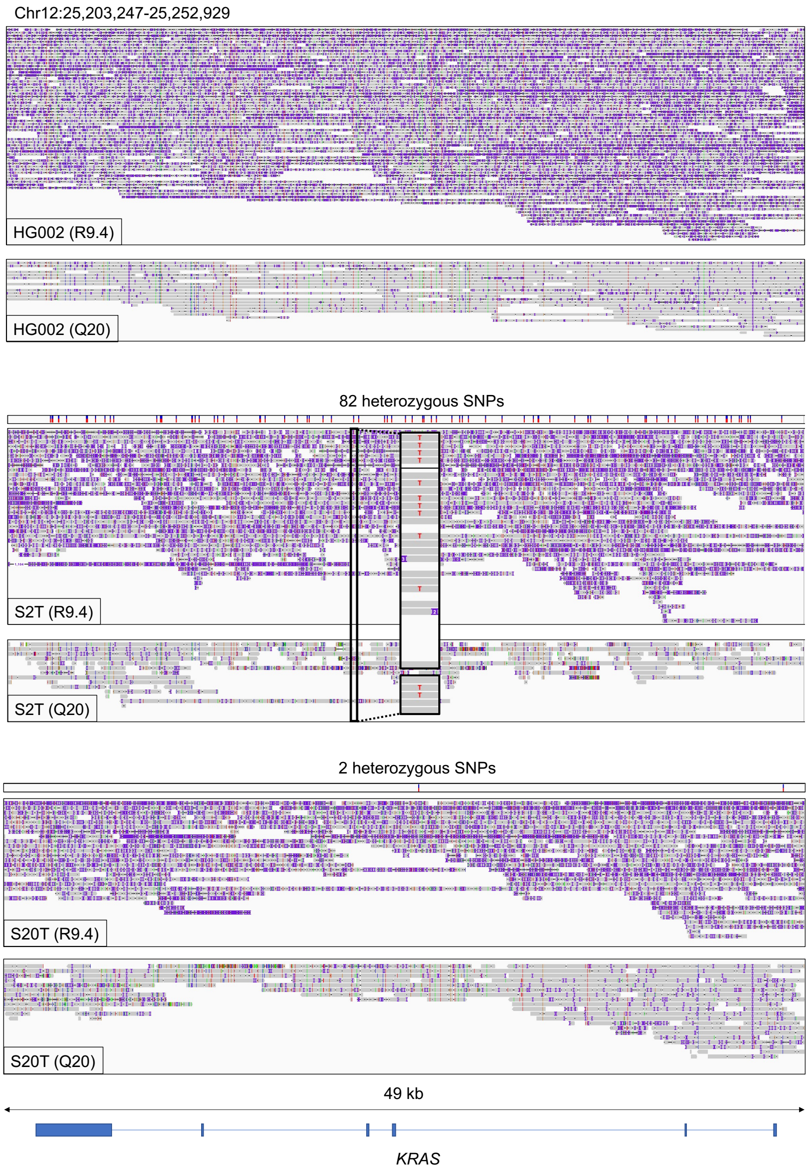
Chr12:25,203,247-25,252,929
HG002 (R9.4)
HG002 (Q20)
82 heterozygous SNPs
S2T (R9.4)
S2T (Q20)
2 heterozygous SNPs
S20T (R9.4)
S20T (Q20)
49 kb
KRAS

Supplementary Figure S3 Evaluation of the PromethION Q20 platform and validation of phase information

(**a**) Read length distribution (upper) and sequence identity (lower) are shown for PromethION Q20 datasets of HG002 (left), S20-T (middle), and S2-T (right). (**b**) Visualization of the PromethION Q20 (lower) and current R9.4 (upper) reads. For three specimens, a *KRAS* region was visualized on IGV.

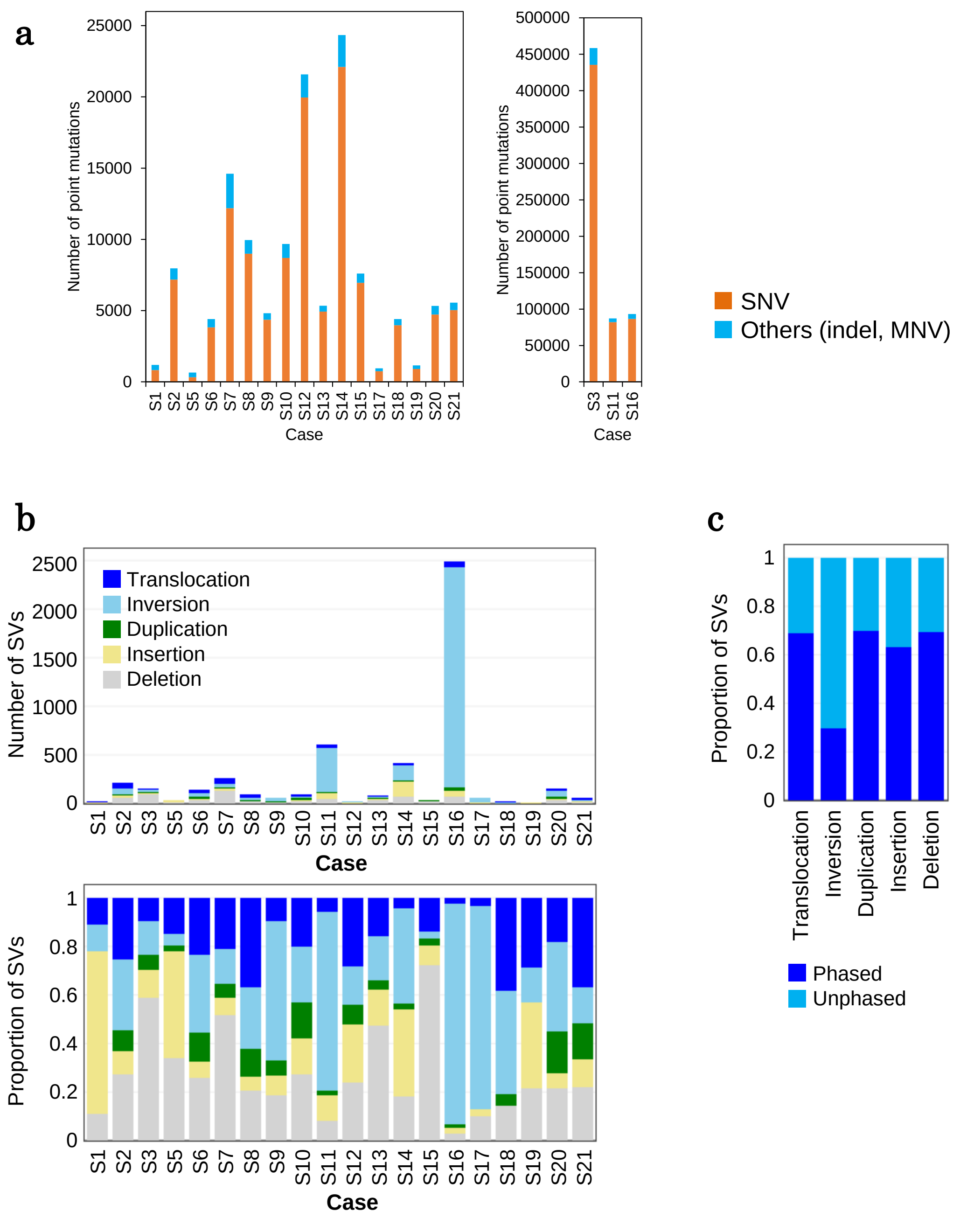

Supplementary Figure S4 The number of somatic mutations in 20 cases

(**a**) The number of point mutations (including SNV, MNV, and indels) in the examined 20 cases. Three cases with numerous mutations are also represented in the right graph. (**b**) The number of SVs in the examined 20 cases. The proportion of SVs for each type is shown in the lower panel. (**c**) The proportion of phased/unphased SVs for each SV type. Source data are provided as a Source Data file for **a**–**c**.

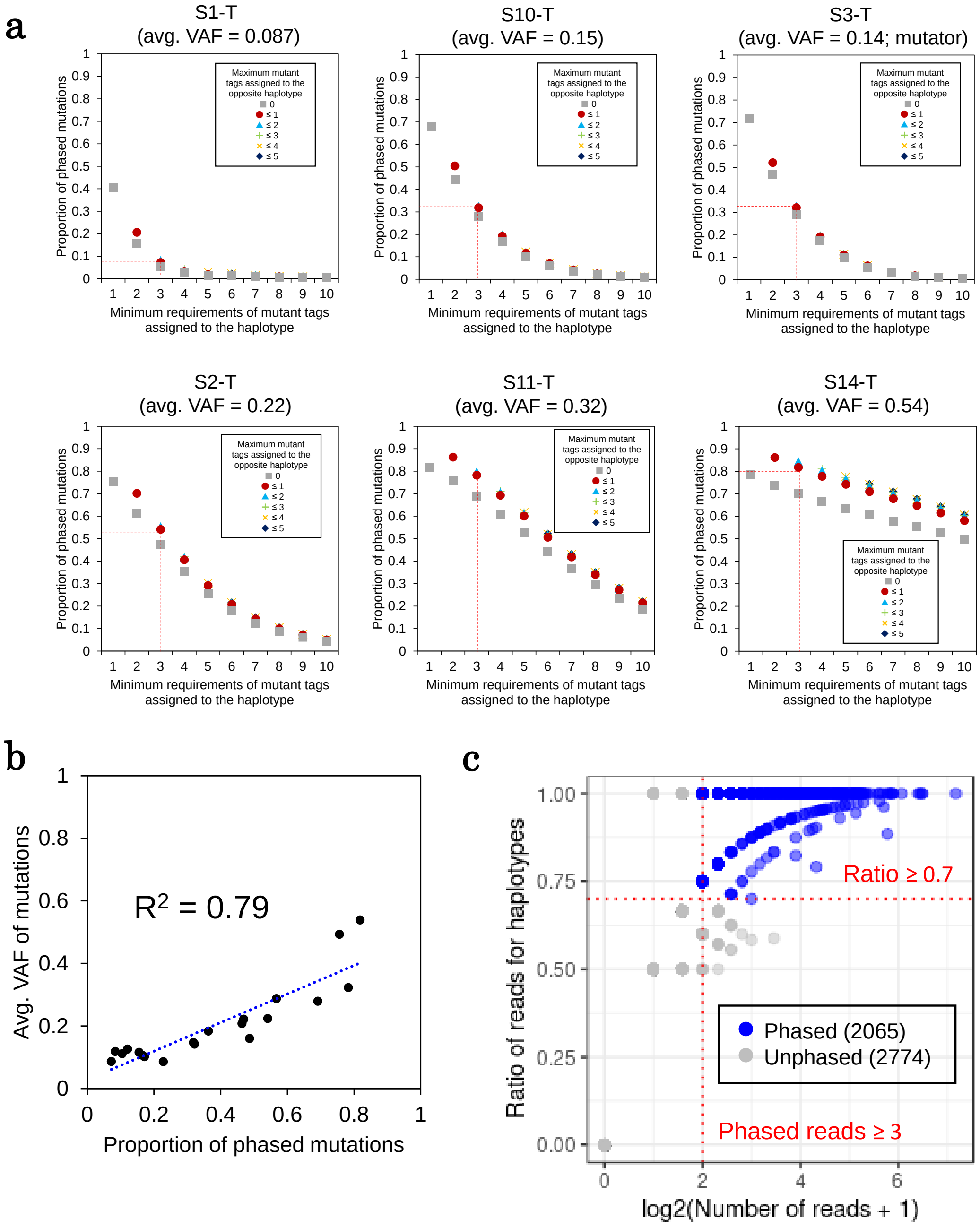

Supplementary Figure S5 Haplotype assignment of point mutations and SVs

(**a**) Proportion of point mutations that were assigned to the haplotypes under each threshold of mutant tag requirements. The x-axis represents the minimum requirements of the mutant reads mapped to one haplotype. The dot shape/color indicates the maximum allowance of the mutant reads observed in the opposite haplotype. We finally decided to assign a haplotype tag to each mutation when three or more mutant reads

were mapped to one haplotype and zero or one mutant read was erroneously observed in the other haplotype. (**b**) The association between the proportion of phased mutations (x-axis) and averages of variant allele frequencies (VAFs) (y-axis) is shown in the graph. Each plot dot represents a case. The linear regression line and R^2 values are presented. (**c**) Thresholds of haplotype assignment of SVs. The x- and y-axis indicate the number of the phased reads and the ratio of the phased reads assigned to one haplotype, respectively. We finally determined "phased" SVs supported with three or more phased reads and the ratio of the reads assigned to HP1 or HP2 being 0.7 or more. Source data are provided as a Source Data file for **b**.

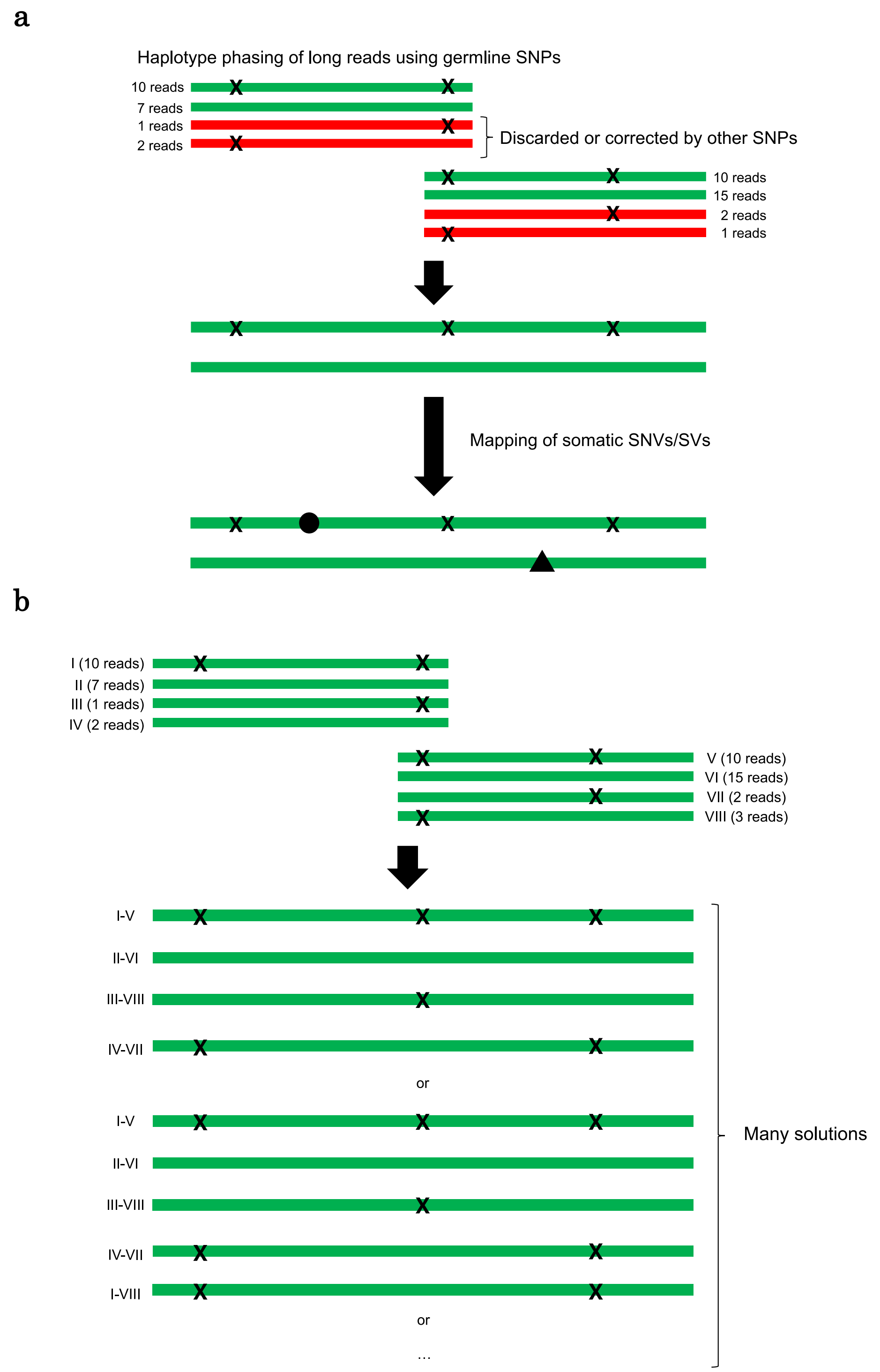

Supplementary Figure S6 Scheme of haplotype phasing

(**a**) Scheme of haplotype phasing and assignment of haplotype number to somatic SNVs/SVs when the number of haplotypes is assumed as two. (**b**) Scheme of haplotype phasing and assignment of haplotype number to somatic SNVs/SVs when the number of haplotypes is ambiguous.

a

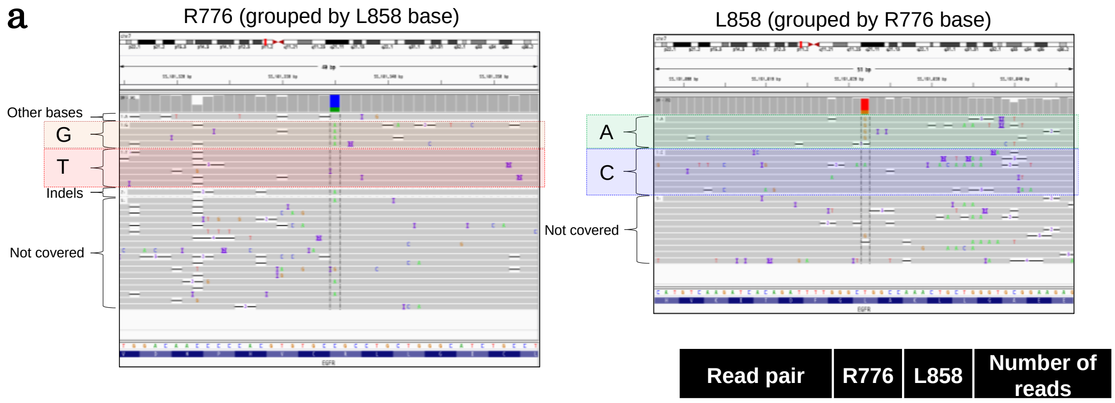

Read pair	R776	L858	Number of reads
Double mutant	A	G	**4**
R776S-L858wt	A	T	0
R776wt-L858R	C	G	0
Both wt	C	T	**6**
Others			2

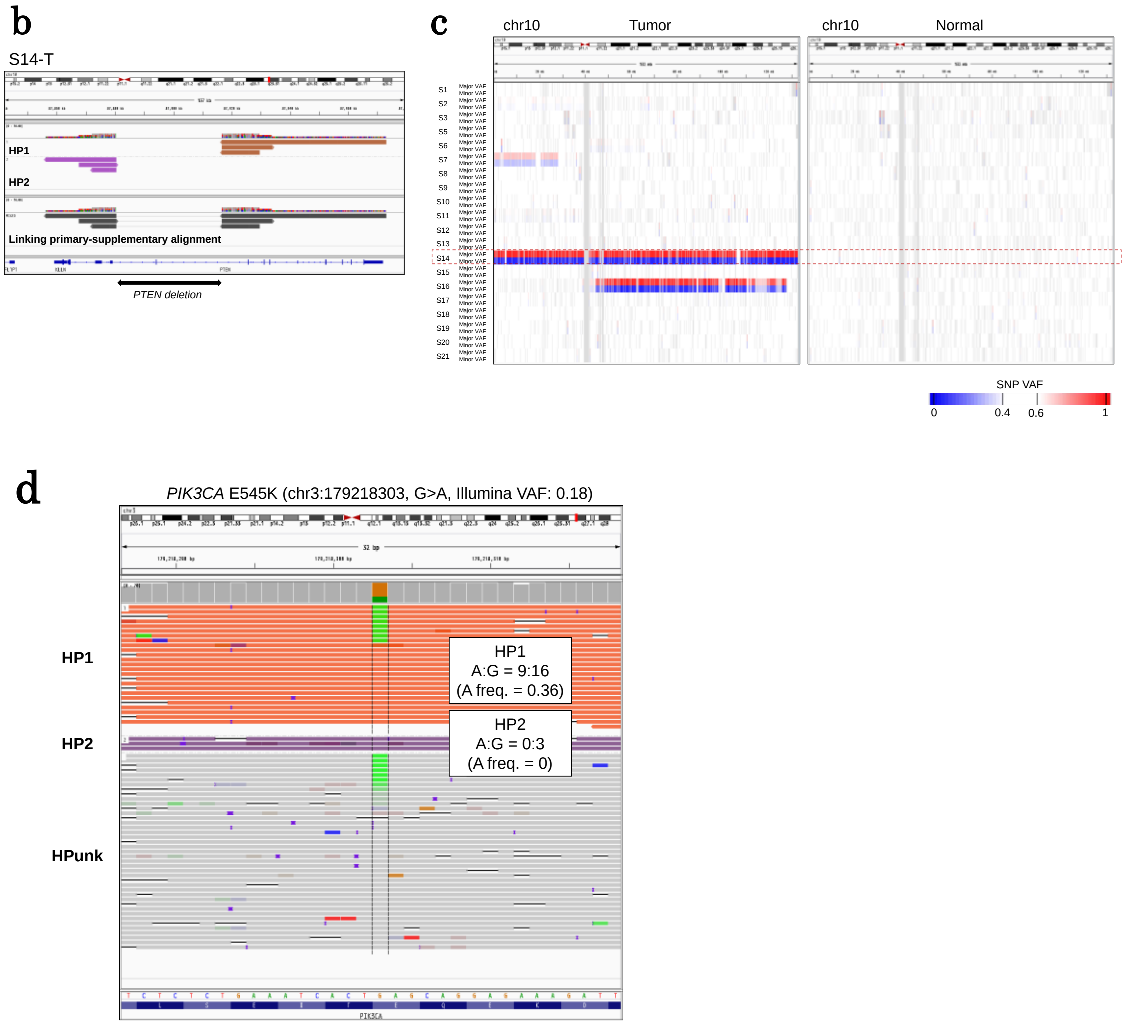

Supplementary Figure S7 Mutations of cancer-related genes at the single-molecule and the haplotype levels

(**a**) The IGV visualization of *EGFR* R776S and L858R mutations in case S21. The PromethION reads were grouped by the L858 or R776 base type. Information on the base combination of these positions is shown at the table at the margin. (**b**) PromethION reads in which the haplotype was resolved in both sides (primary and supplementary alignments) of the breakpoint of *PTEN* deletion are visualized by IGV for case S14. This deletion was located over the two phased blocks. One side was assigned to the HP2 of the upstream block, whereas the other side was assigned to HP1 of the downstream block. (**c**) VAFs of the phase SNPs in each haplotype in chromosome 10. VAFs were calculated using short read WGS and averaged for each phase block. The averaged VAFs were visualized as relative CNs in each haplotype using IGV. For each phase block in each case, SNP VAFs in the haplotypes with larger VAFs ("major VAF") are shown in the upper row, whereas those in the other haplotypes ("minor VAF") are represented in the lower row. A color key is provided at the margin. Panels for tumor and normal specimens are shown in the left and right captions, respectively. (**d**) A *PIK3CA* mutation identified on the PromethION reads is visualized by IGV. The reads are divided according to haplotypes.

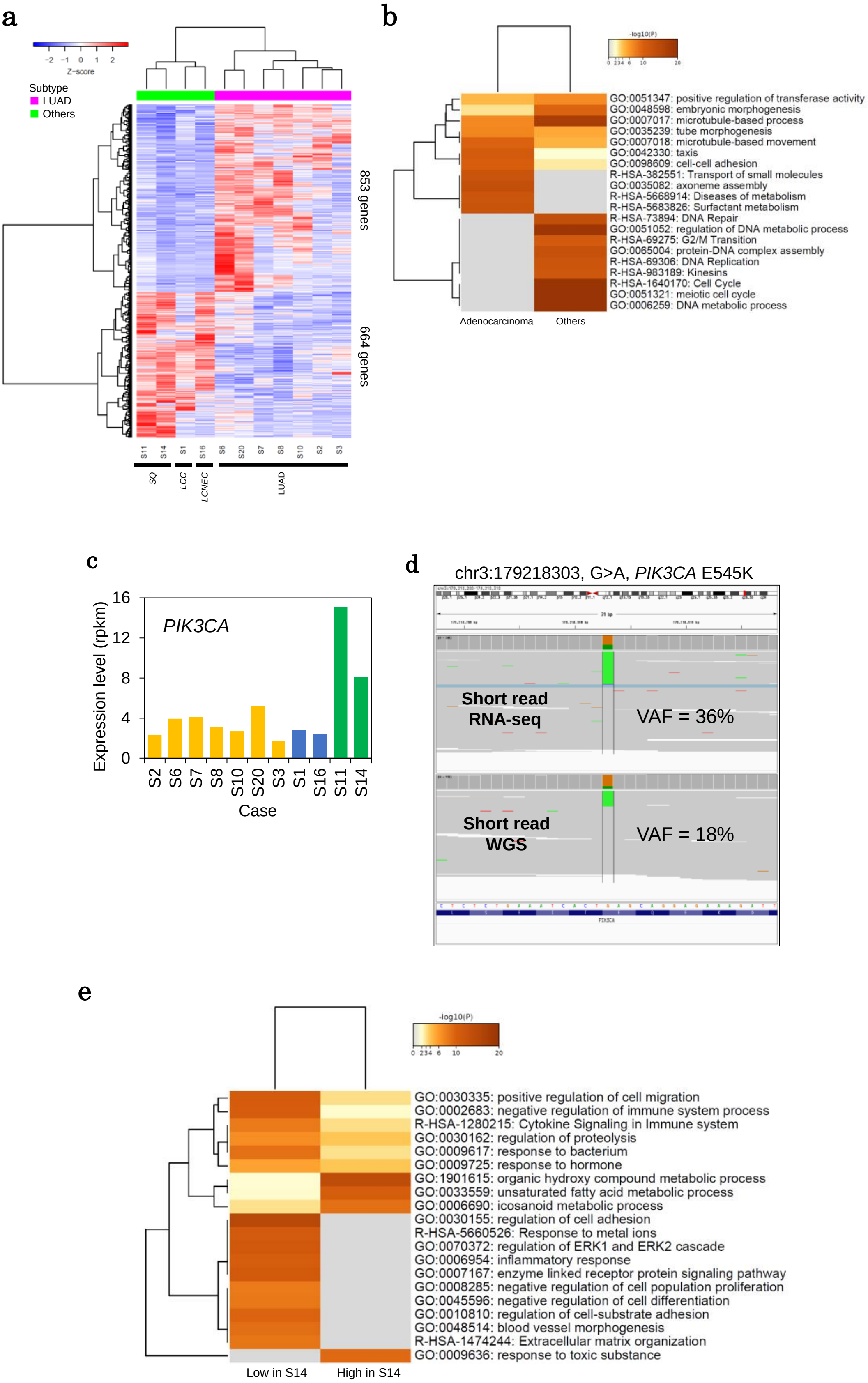
a
Z-score
Subtype
LUAD
Others
853 genes
664 genes
S11
S14
S1
S16
S6
S20
S7
S8
S10
S2
S3
SQ
LCC
LCNEC
LUAD
b
-log10(P)
GO:0051347: positive regulation of transferase activity
GO:0048598: embryonic morphogenesis
GO:0007017: microtubule-based process
GO:0035239: tube morphogenesis
GO:0007018: microtubule-based movement
GO:0042330: taxis
GO:0098609: cell-cell adhesion
R-HSA-382551: Transport of small molecules
GO:0035082: axoneme assembly
R-HSA-5668914: Diseases of metabolism
R-HSA-5683826: Surfactant metabolism
R-HSA-73894: DNA Repair
GO:0051052: regulation of DNA metabolic process
R-HSA-69275: G2/M Transition
GO:0065004: protein-DNA complex assembly
R-HSA-69306: DNA Replication
R-HSA-983189: Kinesins
R-HSA-1640170: Cell Cycle
GO:0051321: meiotic cell cycle
GO:0006259: DNA metabolic process
Adenocarcinoma
Others
c
PIK3CA
Expression level (rpkm)
Case
S2
S6
S7
S8
S10
S20
S3
S1
S16
S11
S14
d
chr3:179218303, G>A, PIK3CA E545K
Short read RNA-seq
VAF = 36%
Short read WGS
VAF = 18%
e
-log10(P)
GO:0030335: positive regulation of cell migration
GO:0002683: negative regulation of immune system process
R-HSA-1280215: Cytokine Signaling in Immune system
GO:0030162: regulation of proteolysis
GO:0009617: response to bacterium
GO:0009725: response to hormone
GO:1901615: organic hydroxy compound metabolic process
GO:0033559: unsaturated fatty acid metabolic process
GO:0006690: icosanoid metabolic process
GO:0030155: regulation of cell adhesion
R-HSA-5660526: Response to metal ions
GO:0070372: regulation of ERK1 and ERK2 cascade
GO:0006954: inflammatory response
GO:0007167: enzyme linked receptor protein signaling pathway
GO:0008285: negative regulation of cell population proliferation
GO:0045596: negative regulation of cell differentiation
GO:0010810: regulation of cell-substrate adhesion
GO:0048514: blood vessel morphogenesis
R-HSA-1474244: Extracellular matrix organization
GO:0009636: response to toxic substance
Low in S14
High in S14

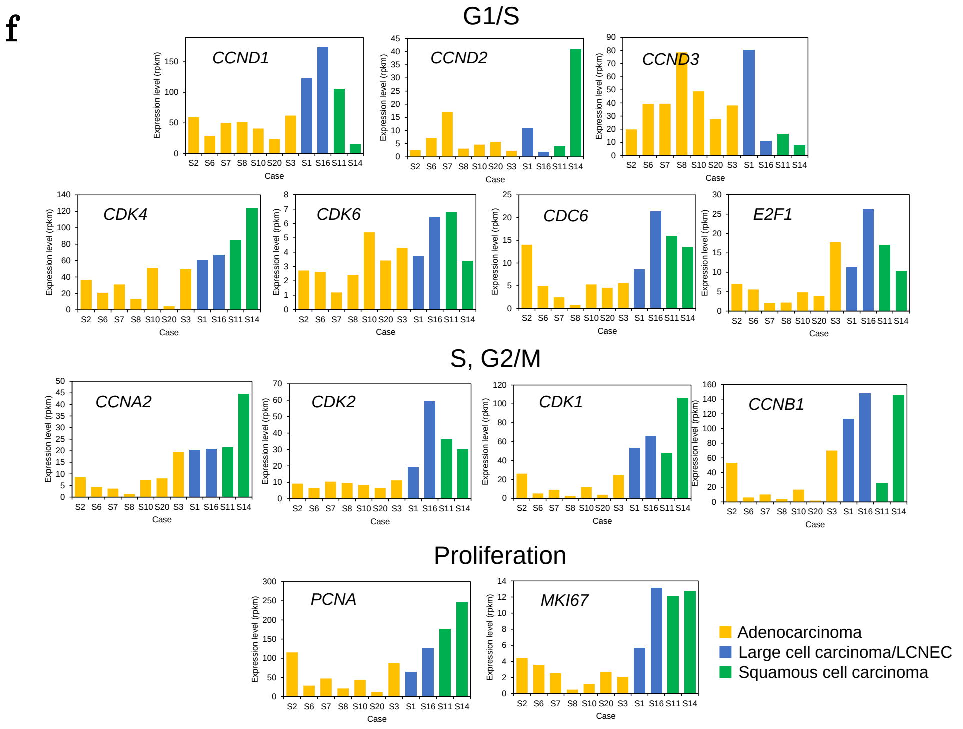

Supplementary Figure S8 RNA-seq analysis for characterizing each case and their characteristic pathways

(**a**) Expression patterns of 11 cases. Differentially expressed genes (DEGs) between lung adenocarcinoma and other subtypes ($p < 0.01$; not adjusted, absolute value of log2 fold change > 1, DESeq2) are represented. AD: adenocarcinoma; SQ: squamous cell carcinoma; LCC: large cell carcinoma; LCNEC: large cell neuroendocrine carcinoma. (**b**) The result of gene enrichment analysis for the DEGs shown in **a**. Enriched term clusters of "Gene Ontology Biological Processes" and "Reactome Gene Sets" (Metascape; https://metascape.org/) are represented. The multi-list enrichment analysis was conducted by Metascape under the default parameters, "Min Overlap = 3", "P Value Cutoff = 0.01", "Min Enrichment = 1.5." (**c**) Expression levels of *PIK3CA* gene. (**d**) The IGV visualization of short read RNA-seq and WGS for the position of the *PIK3CA* mutation in case S14. (**e**) The result of gene enrichment analysis for genes with ≥4-fold or ≤1/4-fold expression between cases S14 and S11 (both from squamous cell carcinoma). Enriched terms of "Gene Ontology Biological Processes" and "Reactome Gene Sets" (Metascape) are represented. The multi-list enrichment analysis was conducted by Metascape under the default parameters, "Min Overlap = 3", "P Value Cutoff = 0.01", "Min Enrichment = 1.5." (**f**) Expression levels of genes associated with cell cycle and cell

proliferation. Source data are provided as a Source Data file.

Note:

We used RNA-seq datasets from 11 cases to characterize pathway activation in each case (**a**). We confirmed that adenocarcinoma cases harbored higher expression levels of "Surfactant metabolism," which included *NAPSA* and several surfactant proteins, that is known to be characteristic in adenocarcinoma (**b**). We further found that other subtypes harbored high expression patterns of cell cycle-related genes compared with adenocarcinoma.

We further closely inspected the gene expression profile of case S14, where a large deletion of the *PTEN* gene and the point mutation of *PIK3CA* were detected (as originally shown in **Figure 3**) and compared it with those of other cases. Particularly, we examined the expression induction of the genes associated with the PTEN/PI3K-AKT pathway, possibly occurring as transcriptional addiction of the pathway activation. First, we could confirm that the *PIK3CA* mutation (E545K) was directly represented in RNA-seq tags, showing that this mutant allele is actively transcribed (**c** and **d**). The expression level of the mutant allele was even higher than the wild-type allele, suggesting that cancer cells in this case should be addicted to the *PIK3CA* mutation. Further, we evaluated the differential expression between cases S14 and S11, which were both derived from the same squamous cell carcinoma (**e**). Case S11 is a *PIK3CA* mutation-negative case and showed the differential expression patterns with the case S14 potentially regarding the PI3K-AKT signaling activation, which might specifically occur in case S14. For example, in case S14, we observed downregulation of "regulation of proteolysis," including *GSK3B*, of which the pathway might be associated with the PI3K-AKT signals. We further inspected the expression levels of cell cycle genes because one of the pivotal phenotypic functions of this pathway is to facilitate cellular proliferation. As expected, we detected that the genes associated with cancer cell proliferation were highly expressed in case S14, which were compatible with those of other cases (**f**). Interestingly, the gene expression level of *CCND2* was particularly high, whereas that of *CCND1* was relatively low in case S14. Analyses of these molecules will reveal details of aberrant signals in which cancer cells would be addicted in a case-specific manner.

a

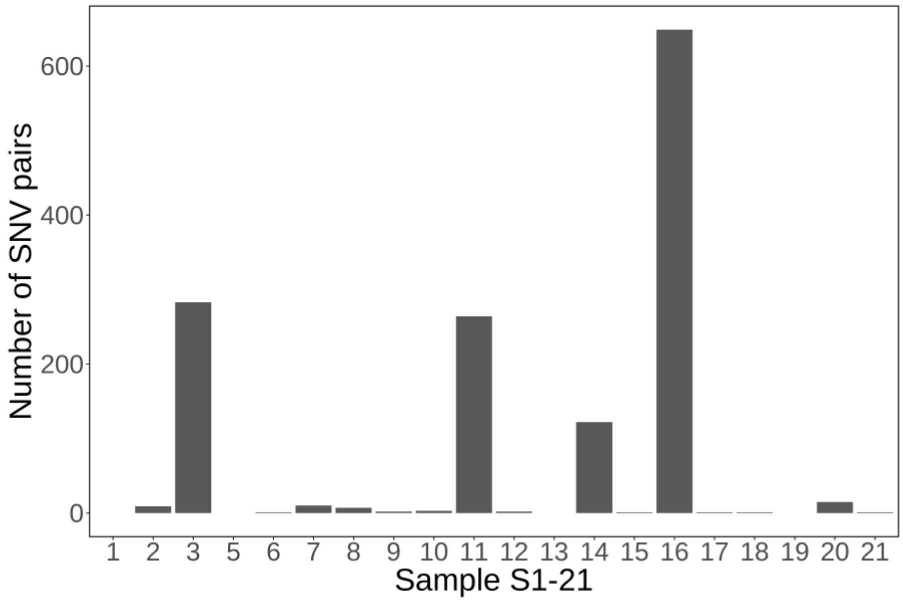
Number of SNV pairs
600
400
200
0
1 2 3 5 6 7 8 9 10 11 12 13 14 15 16 17 18 19 20 21
Sample S1-21

b

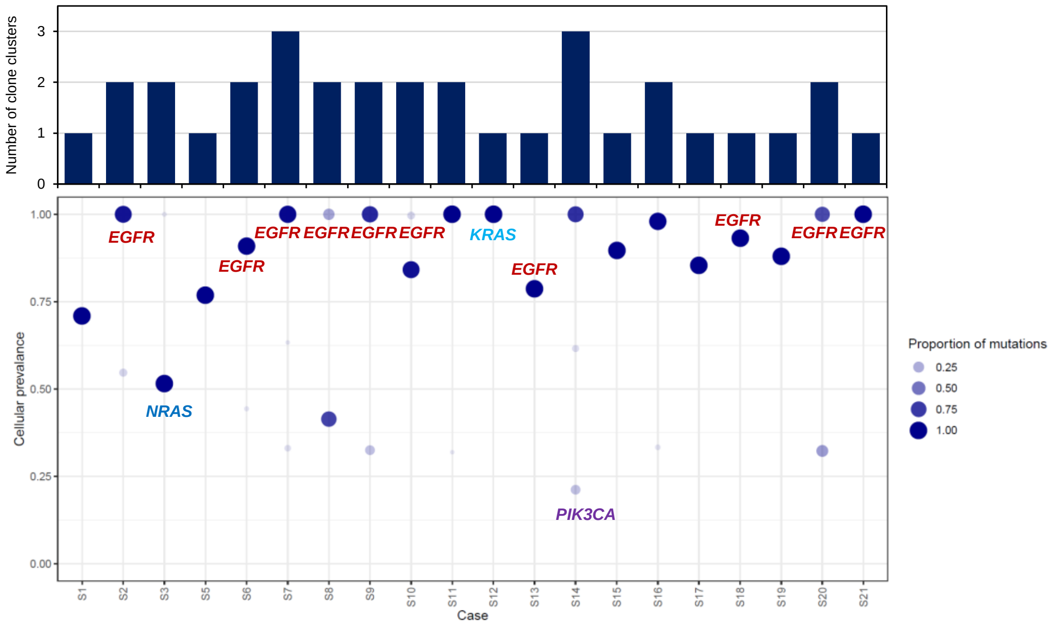
Number of clone clusters
Cellular prevalance
Case
S1 S2 S3 S5 S6 S7 S8 S9 S10 S11 S12 S13 S14 S15 S16 S17 S18 S19 S20 S21
EGFR
NRAS
KRAS
PIK3CA
Proportion of mutations
0.25
0.50
0.75
1.00

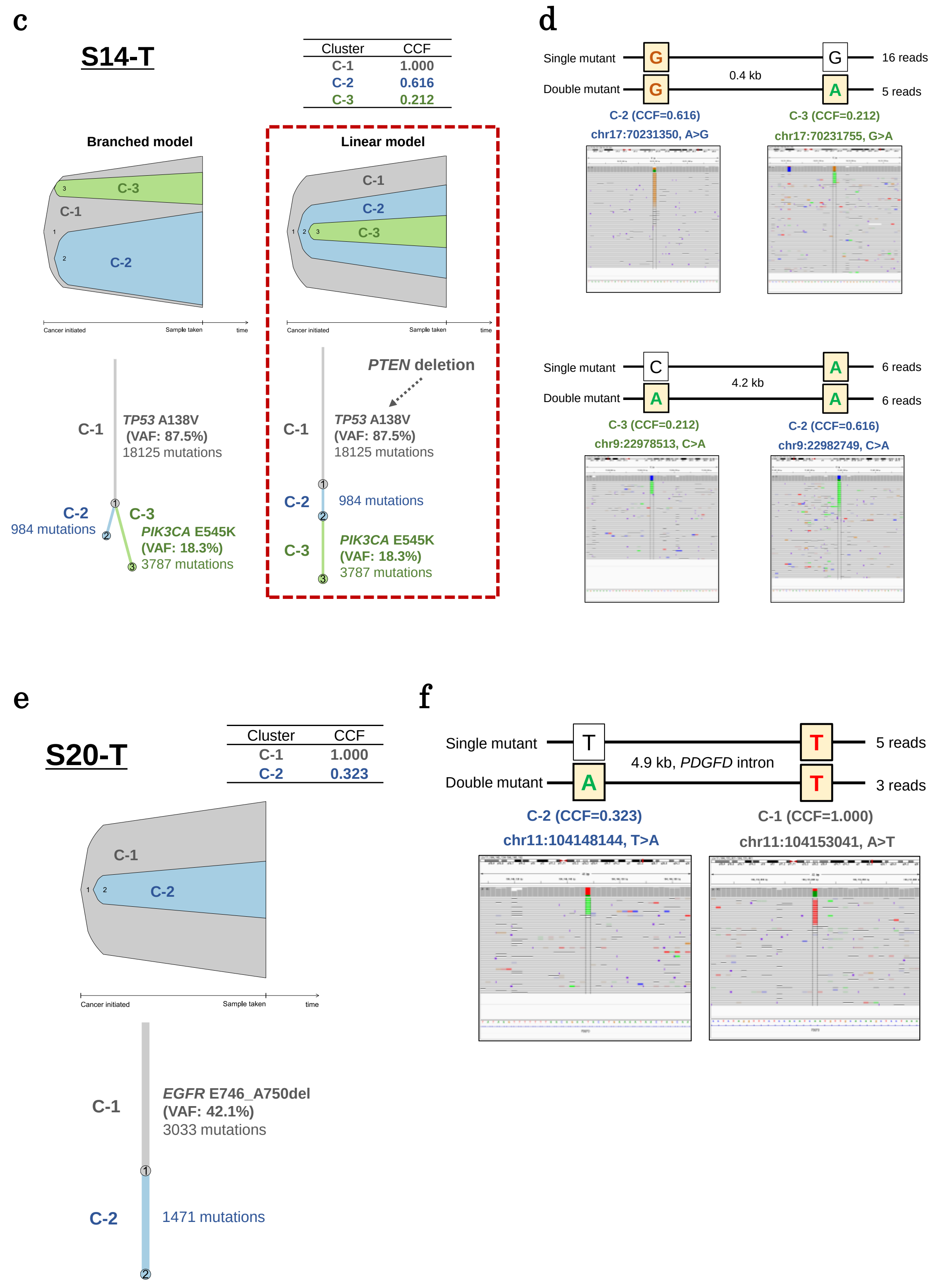

Supplementary Figure S9 Mutation pairs of which the order of occurrence could be

resolved by long reads

(**a**) The number of mutation pairs of which the order of occurrence could be resolved by PromethION reads is shown for each of the examined 20 cases. (**b**) Clones (mutation clusters) detected by PyClone-VI analysis using short read WGS. The number of clones is shown in the bar graph (upper). The cellular prevalence (cancer cell fraction: CCF) of each clone and the proportion of mutations assigned to each clone are also shown in the bottom panel. Driver genes of each case were shown in the inset. (**c**) The clone structure of case S14 inferred by ClonEvol. Bel plots and branch-based trees are shown for two models, the branched and linear models. Information on the number of mutations and the representative mutant genes is shown at the margin. (**d**) The two multiple mutation pairs (assigned to C-2 and C-3) of which the order of occurrence could be resolved by long read sequencing in case S14. (**e**) The clone structure of case S20. A bel plot and branch-based tree are shown for the linear model similarly to **c**. (**f**) An example of mutation pairs of which the order of occurrence could be resolved by long read sequencing in case S20. The order of mutation occurrence agreed with the results of PyClone-VI and ClonEvol analyses. Source data are provided as a Source Data file for **b**.

Note:

For case S14, the linear model (surrounded by a dashed red line in **c**) was selected because the order of two mutation pairs assigned to the structure from C-2 to C-3 is directly supported by long reads (shown in **c**).

a

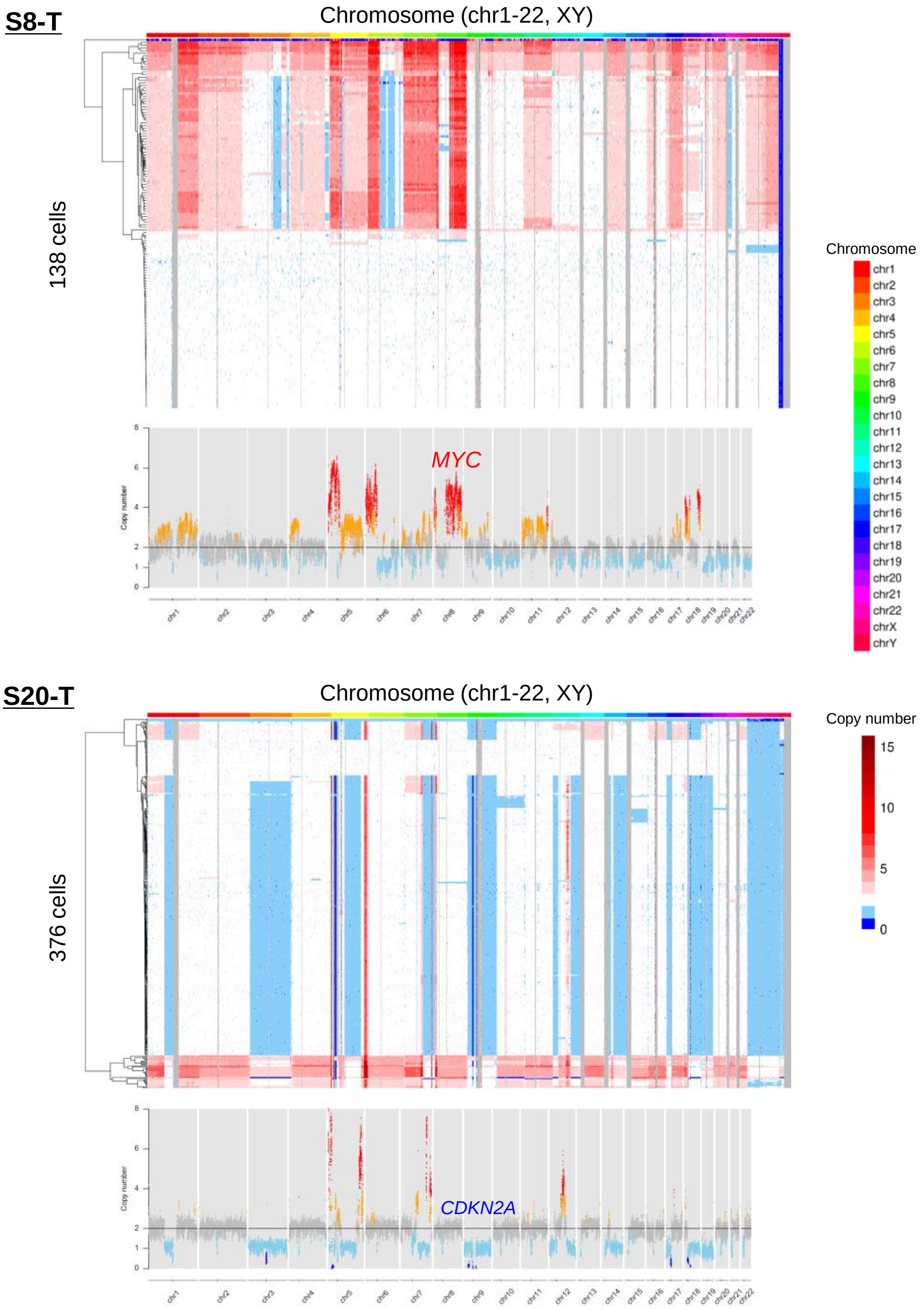

b

	S8		S20	
	Major (C-1)	Minor (C-2)	Major (C-1)	Minor (C-2)
Total (all)	2,750	6,869	3,033	1,471
Covered by scDNA-seq (all)	1,548	1,208	2,886	421
Total (exonic)	32	46	25	12
Covered by scDNA-seq (exonic)	**20**	**4**	**25**	**2**

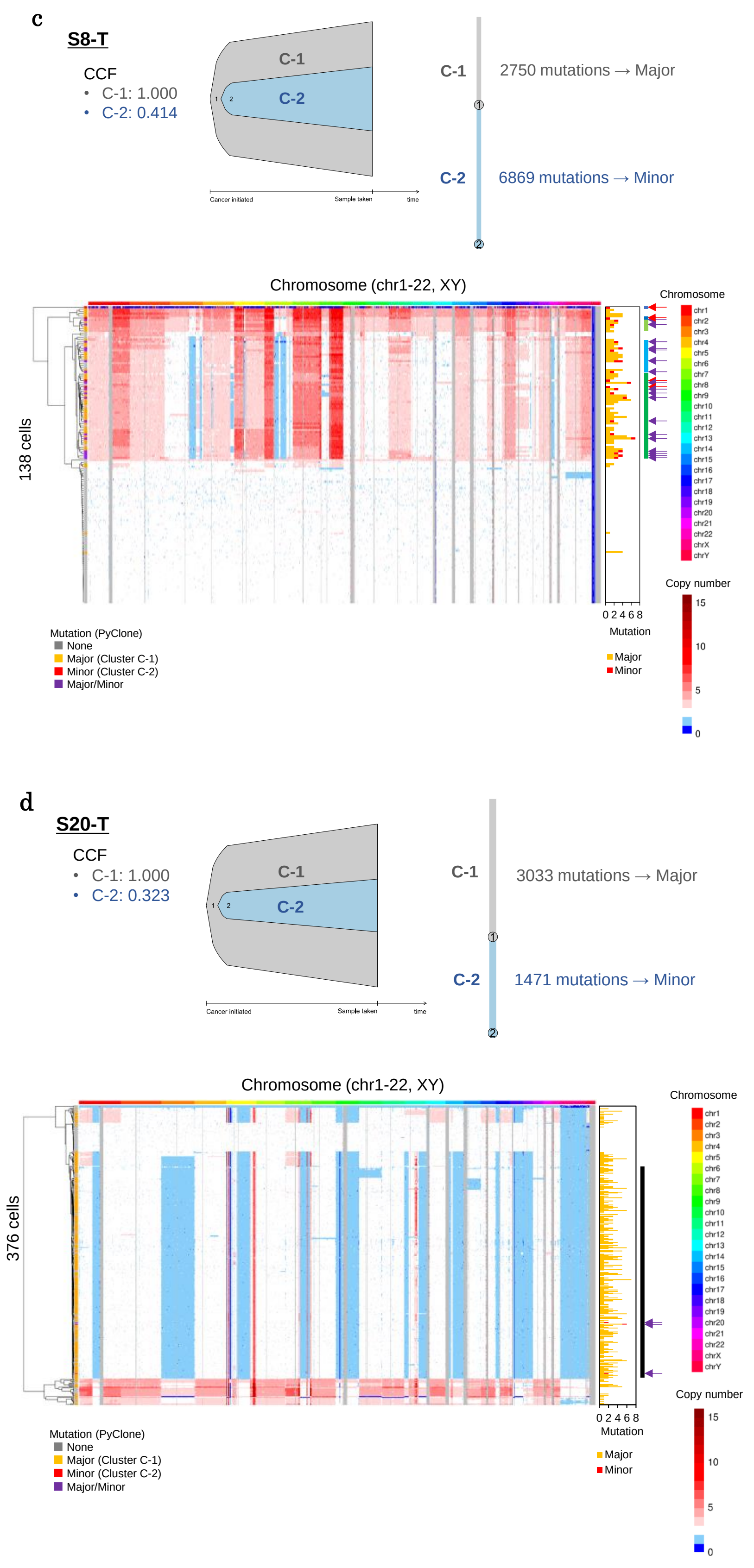
c
S8-T
CCF
• C-1: 1.000
• C-2: 0.414
C-1
C-2
Cancer initiated
Sample taken
time
C-1
2750 mutations → Major
C-2
6869 mutations → Minor
Chromosome (chr1-22, XY)
138 cells
Chromosome
chr1
chr2
chr3
chr4
chr5
chr6
chr7
chr8
chr9
chr10
chr11
chr12
chr13
chr14
chr15
chr16
chr17
chr18
chr19
chr20
chr21
chr22
chrX
chrY
Copy number
15
10
5
0
0 2 4 6 8
Mutation
Major
Minor
Mutation (PyClone)
None
Major (Cluster C-1)
Minor (Cluster C-2)
Major/Minor
d
S20-T
CCF
• C-1: 1.000
• C-2: 0.323
C-1
C-2
Cancer initiated
Sample taken
time
C-1
3033 mutations → Major
C-2
1471 mutations → Minor
Chromosome (chr1-22, XY)
376 cells
Chromosome
chr1
chr2
chr3
chr4
chr5
chr6
chr7
chr8
chr9
chr10
chr11
chr12
chr13
chr14
chr15
chr16
chr17
chr18
chr19
chr20
chr21
chr22
chrX
chrY
Copy number
15
10
5
0
0 2 4 6 8
Mutation
Major
Minor
Mutation (PyClone)
None
Major (Cluster C-1)
Minor (Cluster C-2)
Major/Minor

e

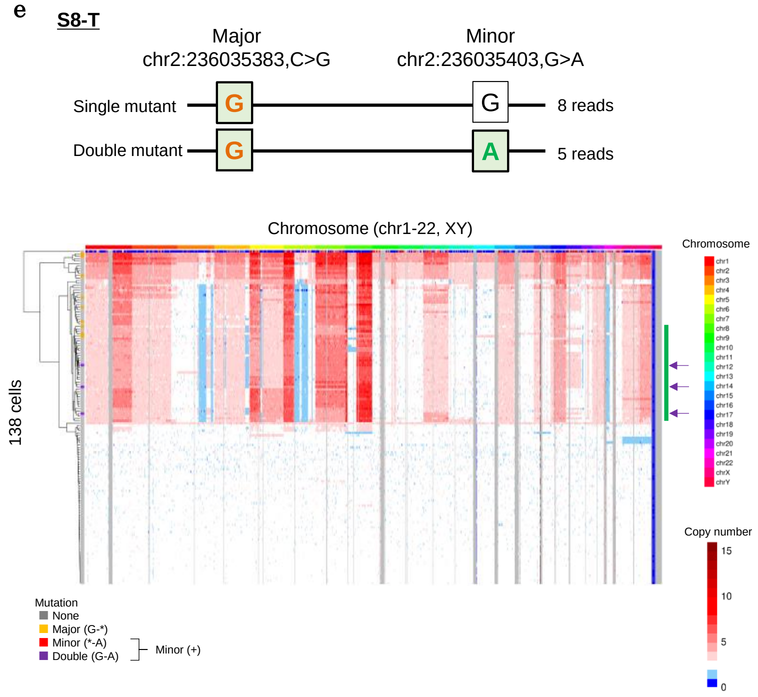

f

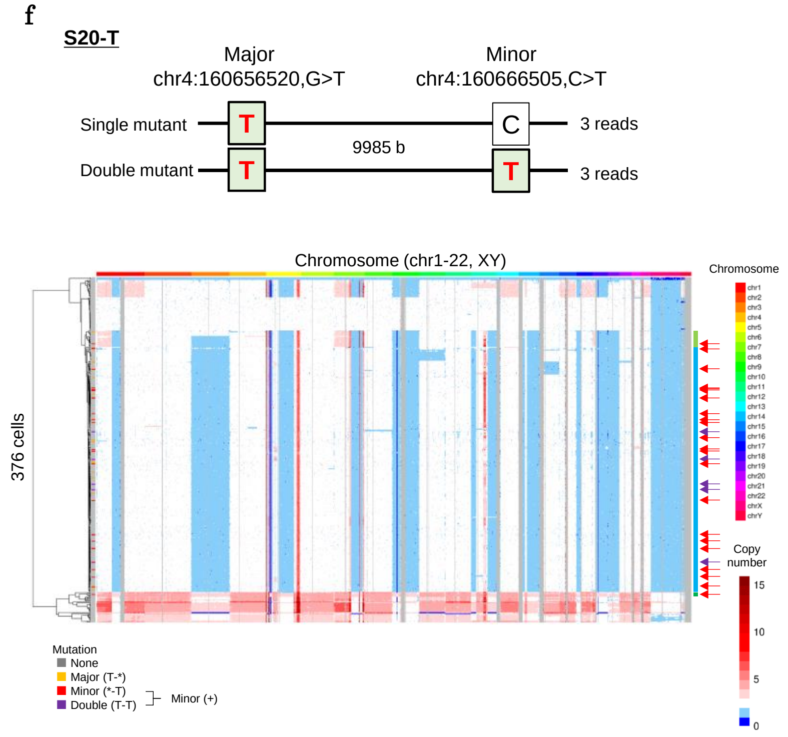

g

	S8-T	S20-T
Total	92	142
Covered by scDNA-seq	**61**	**125**

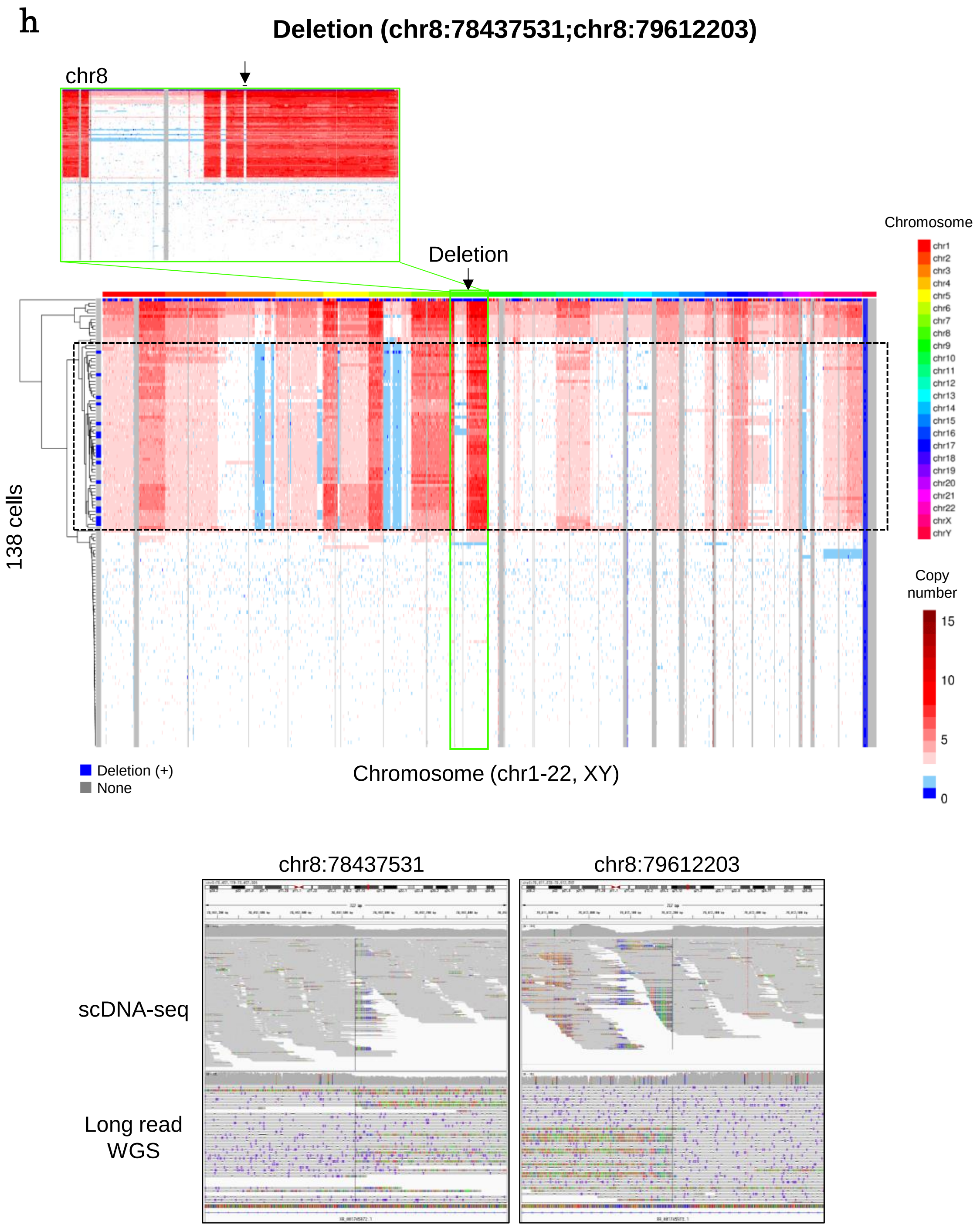
h
Deletion (chr8:78437531;chr8:79612203)
chr8
Deletion
138 cells
Chromosome
chr1
chr2
chr3
chr4
chr5
chr6
chr7
chr8
chr9
chr10
chr11
chr12
chr13
chr14
chr15
chr16
chr17
chr18
chr19
chr20
chr21
chr22
chrX
chrY
Copy number
15
10
5
0
Deletion (+)
None
Chromosome (chr1-22, XY)
chr8:78437531
chr8:79612203
scDNA-seq
Long read WGS

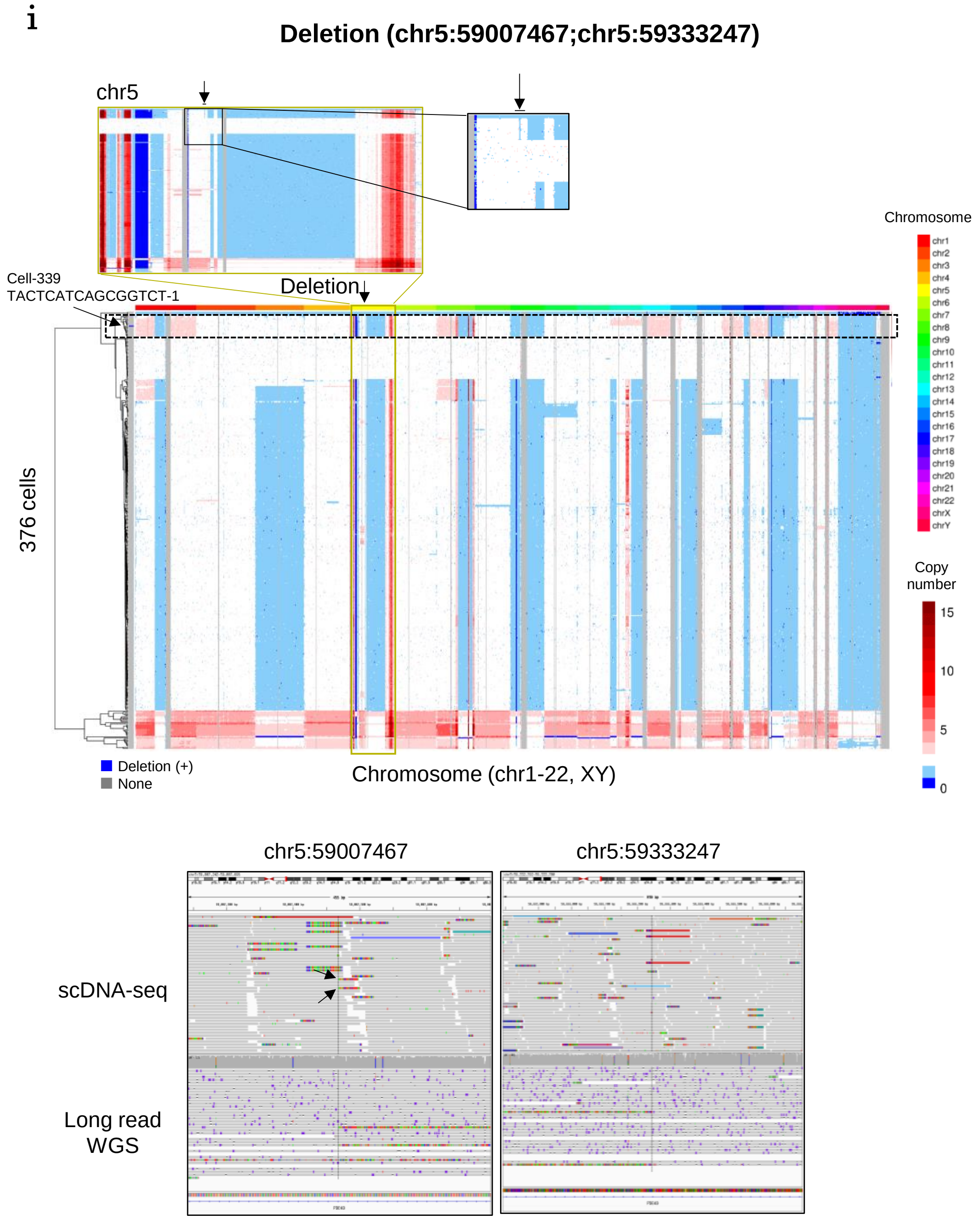

Supplementary Figure S10 Single-cell DNA sequencing (scDNA-seq) of two cases S20 and S8

(**a**) CN profiles from scDNA-seq (upper) and bulk short read WGS (lower) of cases S20 and S8. Clustering analyses of single-cell CN profiles are shown using 138 and 376 cells in S20 and S8, respectively. Bulk CN profiles were calculated by FREEC. (**b**) The table for the total number of point mutations (all and exonic) and the number of mutations covered by scDNA-seq reads. In cases S8 and S20, PyClone analysis classified the

mutations into two clusters, C-1 and C-2. The C-1 and C-2 mutations are defined as "Major" or "Minor" mutations, respectively. (**c**) The clonal structure in case S8 is inferred by PyClone-VI from VAFs of bulk short read WGS data (upper). Single-cell CN profiles are represented in a heatmap with "Major" and "Minor" mutation profiles (lower). The number of mutations detected in each cell is shown in the bar graph. "Minor" mutation-positive cells are highlighted by arrows. (**d**) The clonal structure inferred by PyClone analysis and single-cell CN profiles in case S20. The analysis and visualization of the results are performed similarly as **c**. (**e f**) Examples of mutation pairs of which the order of occurrence is directly resolved by long reads are shown (upper). The mutation in both single and double mutants is defined as "Major," and the other was assigned to the "Minor" mutation. Single-cell CN profiles are represented in a heatmap with "Major" and "Minor" mutation profiles (lower). "Minor" mutation-positive cells are highlighted by arrows. (**g**) The table for the total number of SVs and the number of SVs also covered by scDNA-seq reads. (**h i**) Single-cell CN profiles with the SV status (upper). The corresponding SV region is zoom-in at the margin. The IGV visualization of the SV junctions of scDNA-seq and bulk long read WGS.

Note:
We performed scDNA-seq analysis for two representative cases, cases S8 and S20 (**a**). We first mapped point mutations to individual cells by examining whether the mutant reads were found in the scDNA-seq data of each cell. A substantial number of mutations are represented in the scDNA-seq reads (**b**). Notably, mutations, especially "minor" mutations (cluster C-2) were not always represented (**c** and **d**). Given the limited sequencing coverage and due to the possible "allele-drop" (experimental loss of just two chromosomes within a single cell), a particular number of cells should be collectively analyzed to identify and characterize point mutations and SVs.

We further inspected cases of mutation pairs for which their occurrence orders were directly resolved by the long reads. Two and thirteen mutation pairs were also covered in individual cells in cases S8 and S20, respectively. Two of the examples are shown in **e** and **f**. The "minor" mutation in the mutation pair shown in **e** was detected in a subpopulation of cells, indicating that this "minor" mutation occurred relatively later. Moreover, the "minor" mutation in **f** was separately distributed to subclusters of cells. This mutation might have occurred before the occurrence of CN aberrations comprising the subclusters.

We could further map SVs detected from long read WGS to scDNA-seq reads in **g–i**.

a

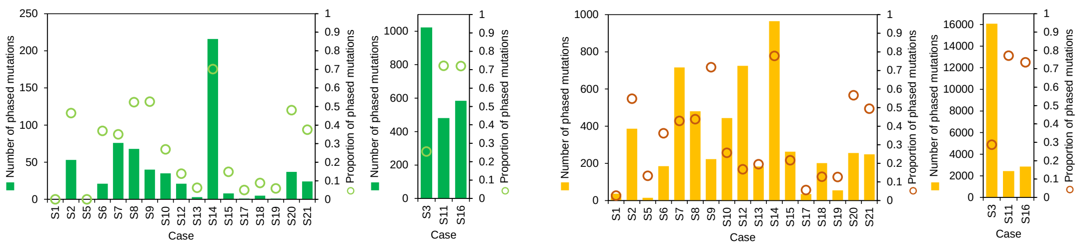

b

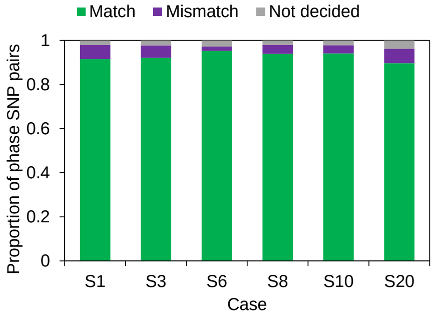

	S1	S3	S6	S8	S10	S20
Match	20,755	31,489	2,735	36,758	22,322	5,664
Mismatch	1,442	1,943	56	1,592	875	416
Not decided	475	754	80	794	512	236

c

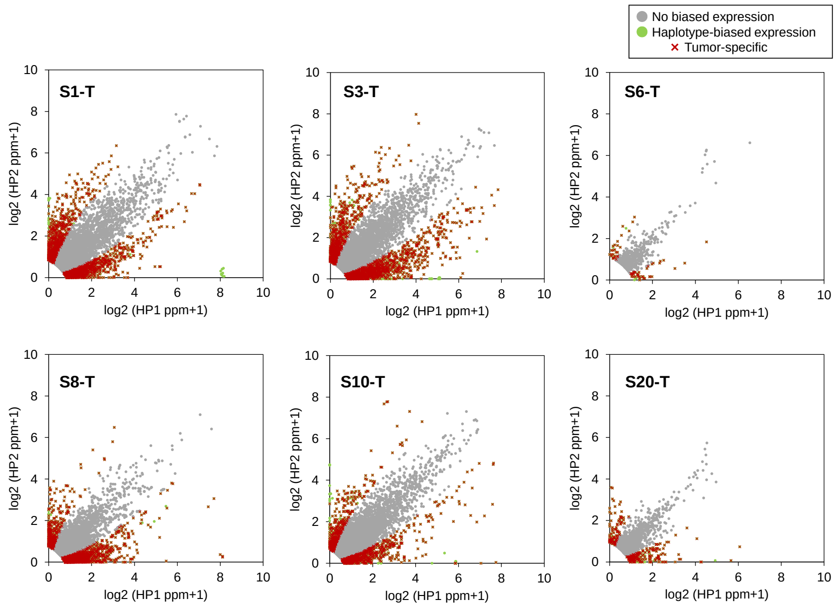

Number of SNPs	S1	S3	S6	S8	S10	S20
All expressed (≥20 tags)	5814	6551	640	5354	7994	1885
Haplotype-biased expression (≥4-fold difference among haplotype)	1782	2266	71	3661	1431	335
Tumor-specific biased expression (Non-bias in normal sample)	1704 (29%)	2114 (32%)	65 (10%)	1604 (30%)	1307 (16%)	313 (17%)

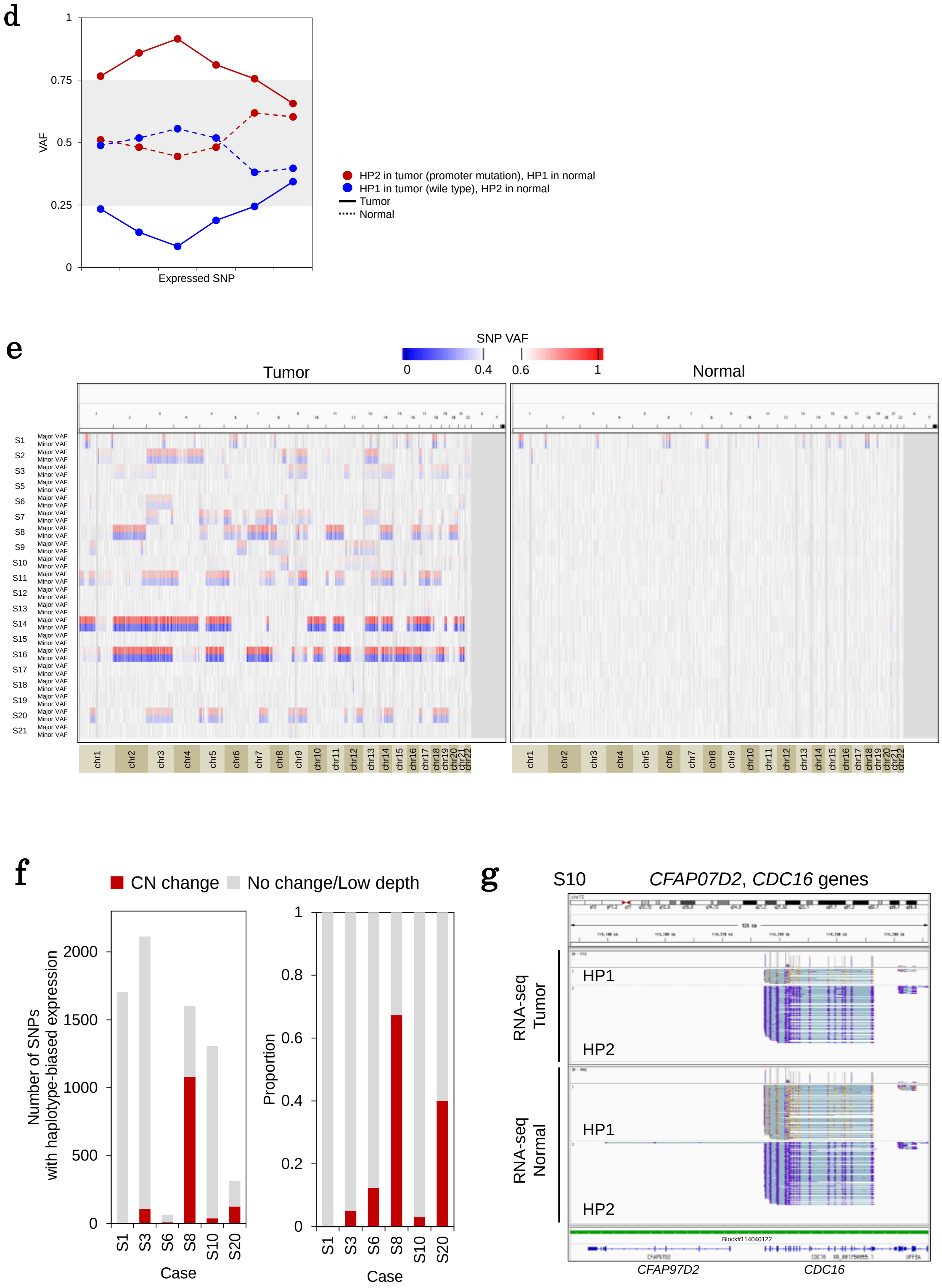

Supplementary Figure S11 Transcriptional regulation bias in cancer genomes

(a) Number of haplotype-resolved mutations in the promoter (upper) and enhancer

(lower) regions. (**b**) Concordance of phase information in the long read RNA-seq data compared with those from WGS Whatshap. The proportion of the SNP pairs of which the phase information was the same (match), different (mismatch) or undetermined (not decided) in the RNA-seq data is shown in the graph (left). The number of SNP pairs in each breakdown is presented in the table (right). (**c**) Expression levels of each of the two haplotypes (HP1 and HP2) in six cases (upper). Each dot represents phased SNPs (≥20 short read RNA-seq tags). The differential expression between haplotypes (≥4-fold expression changes) is depicted in pink. Among them, SNPs with tumor-specific biased expression (<2-fold expression changes between haplotypes in normal RNA-seq data) are also shown with cross marks in red. The number of phased SNPs is presented in the table (lower). (**d**) VAFs of the short read RNA-seq for six SNPs in the *CLN5* exonic region (related to **Figure 4b**). (**e**) Genome-wide VAFs of phase SNPs in each haplotype for inspecting relative CNs in each haplotype. VAFs were calculated and visualized similarly as in **Supplementary Figure S7c**. (**f**) Association between haplotype-biased expression and relative genomic CN changes. The number of SNPs with differential expression between haplotypes calculated in **c**. A proportion of the biasedly transcribed SNPs with the relative CN changes is shown in graphs. (**g**) Transcription patterns of the downstream of the differential methylated regions between haplotypes (shown in **Figure 4e**). Expression levels of *CCDC16* were relatively upregulated in the HP2 of the tumor specimen. Source data are provided as a Source Data file for **a**, **d** and **f**.

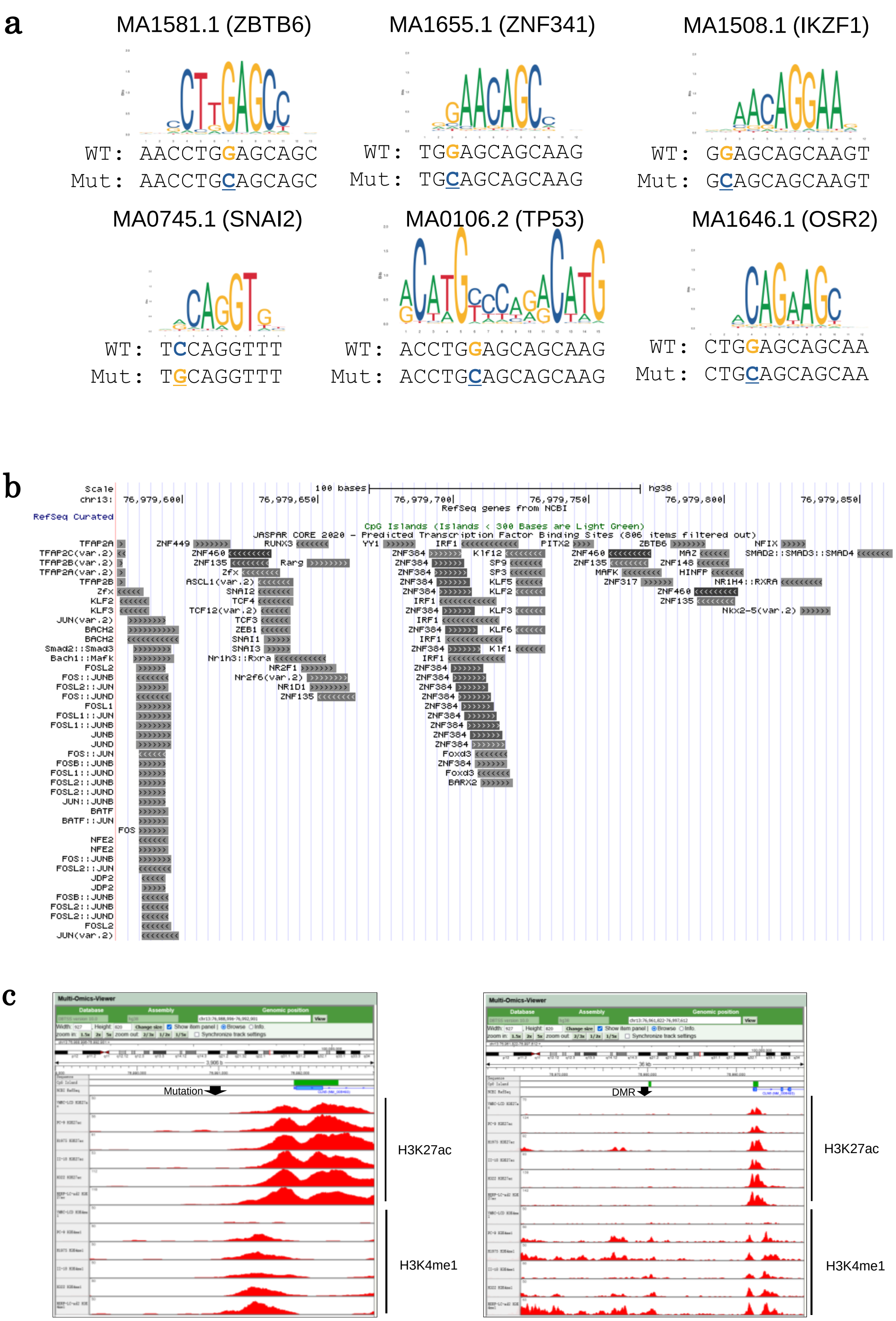
a
MA1581.1 (ZBTB6)
WT: AACCTGGAGCAGC
Mut: AACCTGCAGCAGC
MA1655.1 (ZNF341)
WT: TGGAGCAGCAAG
Mut: TGCAGCAGCAAG
MA1508.1 (IKZF1)
WT: GGAGCAGCAAGT
Mut: GCAGCAGCAAGT
MA0745.1 (SNAI2)
WT: TCCAGGTTT
Mut: TGCAGGTTT
MA0106.2 (TP53)
WT: ACCTGGAGCAGCAAG
Mut: ACCTGCAGCAGCAAG
MA1646.1 (OSR2)
WT: CTGGAGCAGCAA
Mut: CTGCAGCAGCAA
b
Scale
chr13:
100 bases
hg38
76,979,600
76,979,650
76,979,700
76,979,750
76,979,800
76,979,850
RefSeq genes from NCBI
RefSeq Curated
CpG Islands (Islands < 300 Bases are Light Green)
JASPAR CORE 2020 - Predicted Transcription Factor Binding Sites (806 items filtered out)
TFAP2A
TFAP2C(var.2)
TFAP2B(var.2)
TFAP2A(var.2)
TFAP2B
Zfx
KLF2
KLF3
JUN(var.2)
BACH2
BACH2
Smad2::Smad3
Bach1::Mafk
FOSL2
FOS::JUNB
FOSL2::JUN
FOS::JUND
FOSL1
FOSL1::JUN
FOSL1::JUNB
JUNB
JUND
FOS::JUN
FOSB::JUNB
FOSL1::JUND
FOSL2::JUNB
FOSL2::JUND
JUN::JUNB
BATF
BATF::JUN
FOS
NFE2
NFE2
FOS::JUNB
FOSL2::JUN
JDP2
JDP2
FOSB::JUNB
FOSL2::JUNB
FOSL2::JUND
FOSL2
JUN(var.2)
ZNF449
RUNX3
ZNF460
ZNF135
Rarg
Zfx
ASCL1(var.2)
SNAI2
TCF4
TCF12(var.2)
TCF3
ZEB1
SNAI1
SNAI3
Nr1h3::Rxra
NR2F1
Nr2f6(var.2)
NR1D1
ZNF135
YY1
IRF1
ZNF384
Klf12
SP9
SP3
KLF5
KLF2
KLF3
KLF6
Klf1
Foxd3
BARX2
PITX2
ZBTB6
NFIX
ZNF460
MAZ
SMAD2::SMAD3::SMAD4
ZNF135
ZNF148
MAFK
HINFP
ZNF317
NR1H4::RXRA
Nkx2-5(var.2)
c
Multi-Omics-Viewer
Mutation
H3K27ac
H3K4me1
Multi-Omics-Viewer
DMR
H3K27ac
H3K4me1

Supplementary Figure S12 The TFBS candidates in regulatory regions of *CLN5* in case S10

(**a**) Representative candidates of TFBSs on the ±10-bp region of the promoter mutation of the *CLN5* gene. The list is presented in **Supplementary Table S10**. The binding scores of ZBTB6, ZNF341 and IKZF1 decreased with the mutation, whereas those of SNAI2, TP53, and OSR2 increased in the mutant sequence. Sequence logos of each TFBS matrix were provided from the database JASPAR 2022 (https://jaspar.genereg.net/). (**b**) The distribution of TFBS candidates on the DMR was represented by the UCSC Genome Browser (GRCh38/hg38) with JASPAR 2020 (Score: 400). (**c**) H3K27ac and H3K4me1 (enhancer marks) ChIP-seq data of representative lung cancer cell lines are shown in the surrounding region of the mutation (left) and DMR (right). The data are shown in the database DBKERO (https://kero.hgc.jp/)[14].

Note:

In this case, a detected genomic mutation in its promoter region weakened the binding scores of several TFs. The TFBSs included those of ZBTB6 and ZNF341. Moreover, the binding site of SNAI2 was newly created (the binding score was strengthened) by this mutation. *SNAI2* gene is known to be one of the master regulators of epithelial-mesenchymal transition (EMT)[15]. We confirmed the significant level of the gene expression of SNAI2 (10.0 rpkm) in case S10 using RNA-seq data. It is possible that some EMT-related factors, including SNAI2, can regulate the *CLN5* transcription instead of original TF components in the mutant promoter.

We also examined potential TFBSs of the HP2-specific hypomethylated region in the 12-kb upstream of the gene. Several TFBSs of EMT-related factors, including SNAI2 itself, were also detected in this region. We further searched public cell line data for the open chromatin/enhancer regions and found that these two regions both form relevant open chromatin structures. Based on these data, we consider that the detected promoter mutation may "cause" the upstream hypomethylated region and the promoter of the *CLN5* gene to interact more intensively via the binding of SNAI2 and may cooperatively realize the "subsequent" upregulation of the *CLN5* expression.

a

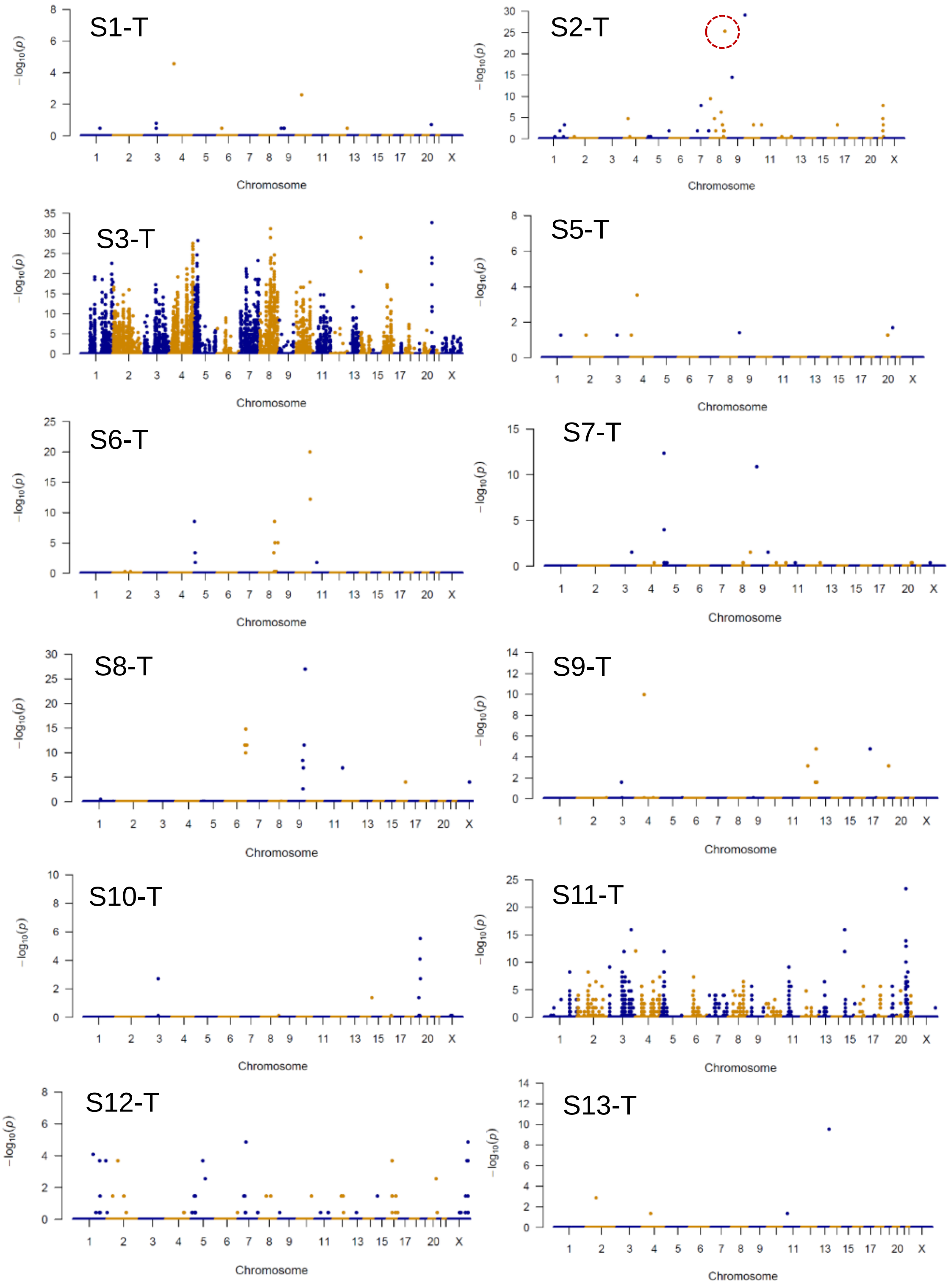

S1-T
S2-T
S3-T
S5-T
S6-T
S7-T
S8-T
S9-T
S10-T
S11-T
S12-T
S13-T
−log10(p)
Chromosome
1 2 3 4 5 6 7 8 9 11 13 15 17 20 X

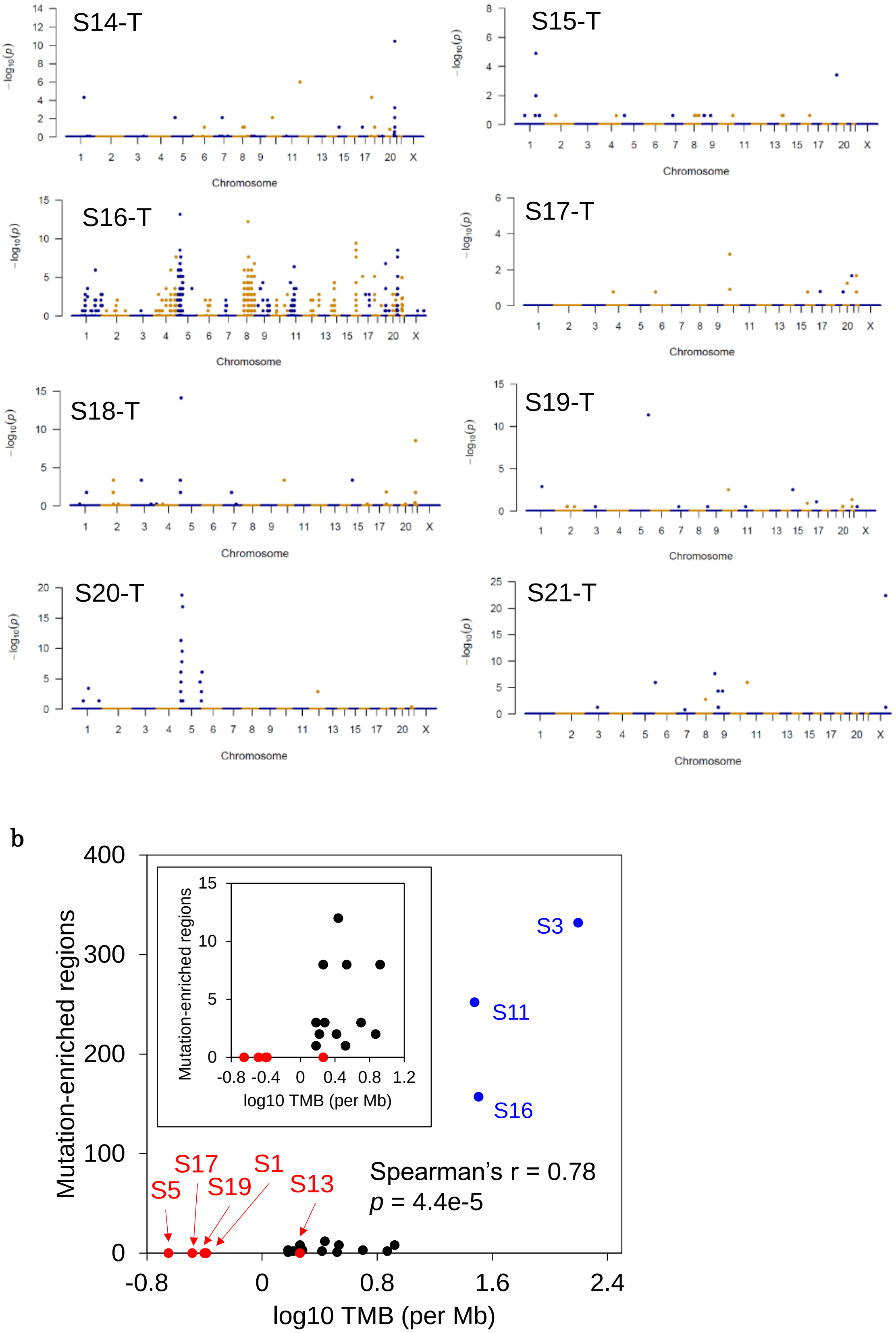
S14-T
S15-T
S16-T
S17-T
S18-T
S19-T
S20-T
S21-T
Chromosome
b
Mutation-enriched regions
log10 TMB (per Mb)
S3
S11
S16
S5
S17
S19
S1
S13
Spearman's r = 0.78
p = 4.4e-5

c

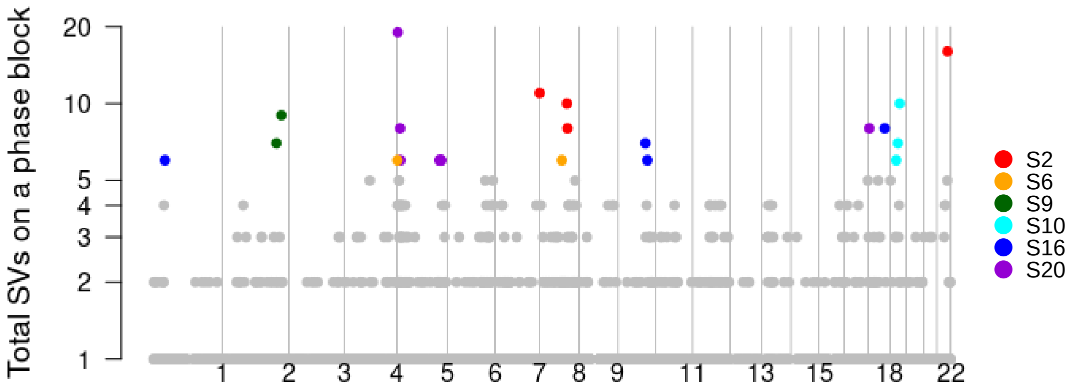

Supplementary Figure S13 Mutation-enriched regions in tumor genomes

(**a**) Genome-wide patterns of mutation-enriched regions for point mutations. The x- and y-axis represent the chromosome positions and Bonferroni-adjusted p-values of enrichment, respectively. In case S2, the plot dot marked by a red circle indicates the region of interest analyzed in **Figure 5c**. (**b**) Association between the number of mutation-enriched blocks and TMB of each case. Each plot represents each case. Five cases with no mutation-enriched blocks are shown in red. The plot without three outliers (blue) is shown in the inset. Spearman's r and p-value (two-sided, no multiple comparison adjustments) are also represented in the inset. (**c**) Genome-wide information about the number of SVs on a phase block in the examined 20 specimens. The phased blocks with ≥6 SVs are shown in the color of each specimen. Source data are provided as a Source Data file for **b**.

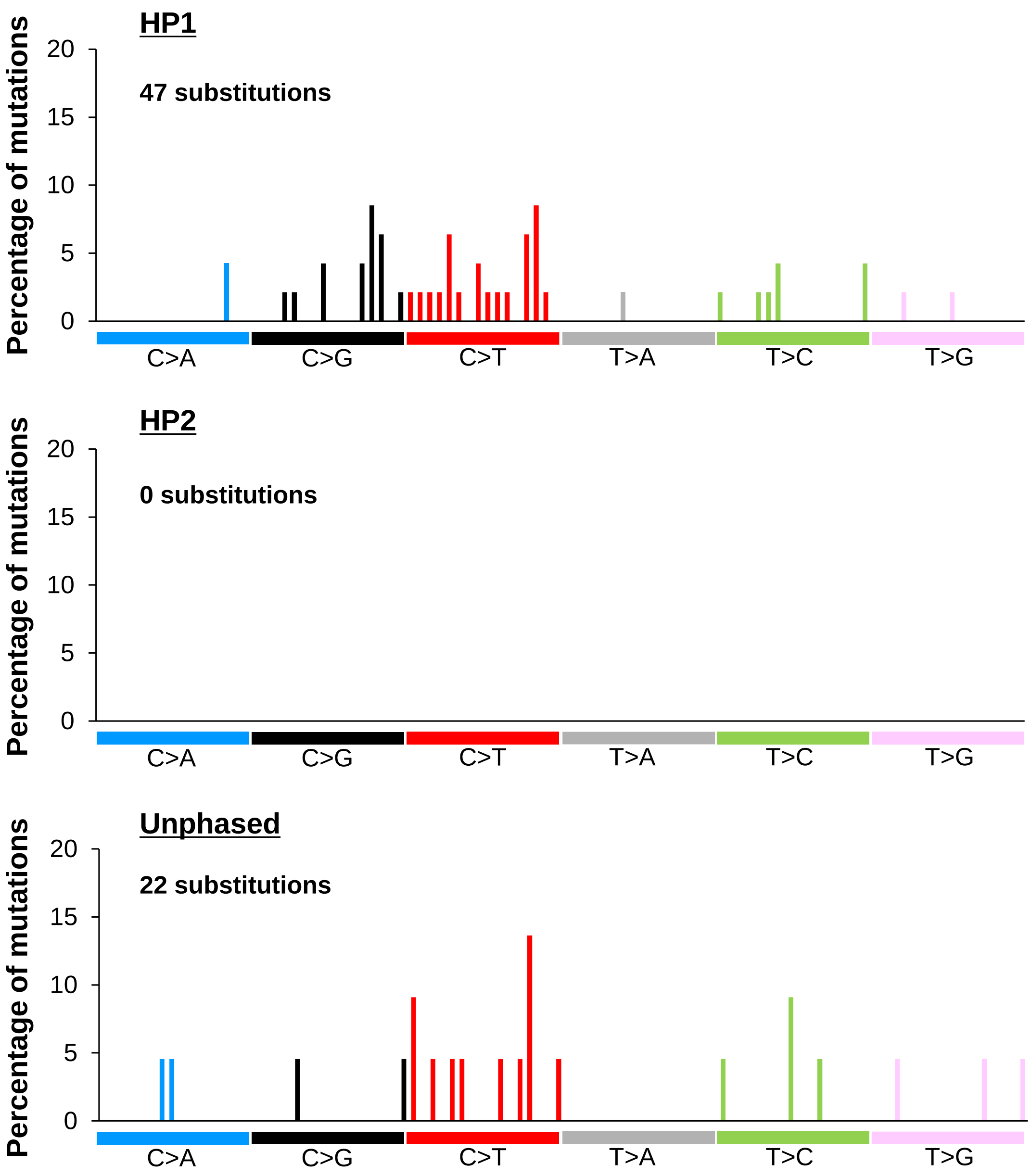

Supplementary Figure S14 Mutational signatures in each haplotype

Summary of 96 mutational patterns in each haplotype on a phase block (chr5: 31,109-3,778,093), as identified in case S20. Source data are provided as a Source Data file.

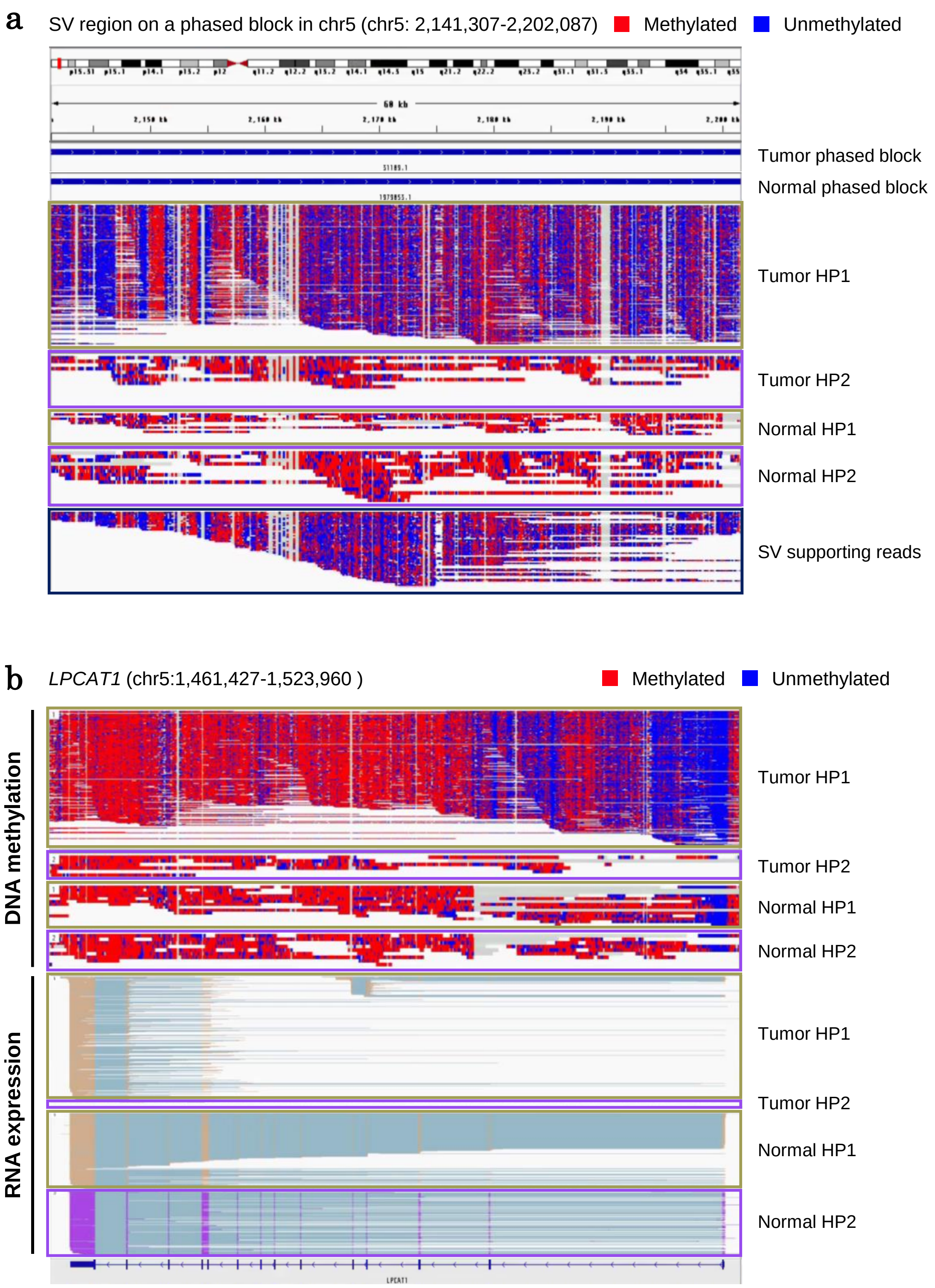

Supplementary Figure S15 Mutation-enriched regions in tumor genomes

(a) Methylation status in the phase block located in the chromothripsis region of case

S20. Normal and tumor panels are presented with reads assigned to each haplotype. The SV panel presents SV-supporting reads detected by Nanomonsv. (**b**) The methylation status and RNA expression level of the *LPCAT1* gene coding region located in the chromothripsis region of case S20. DNA methylation panels summarize reads of the WGS, whereas RNA expression panels summarize reads of the RNA-seq obtained from PromethION; both are assigned to each haplotype.

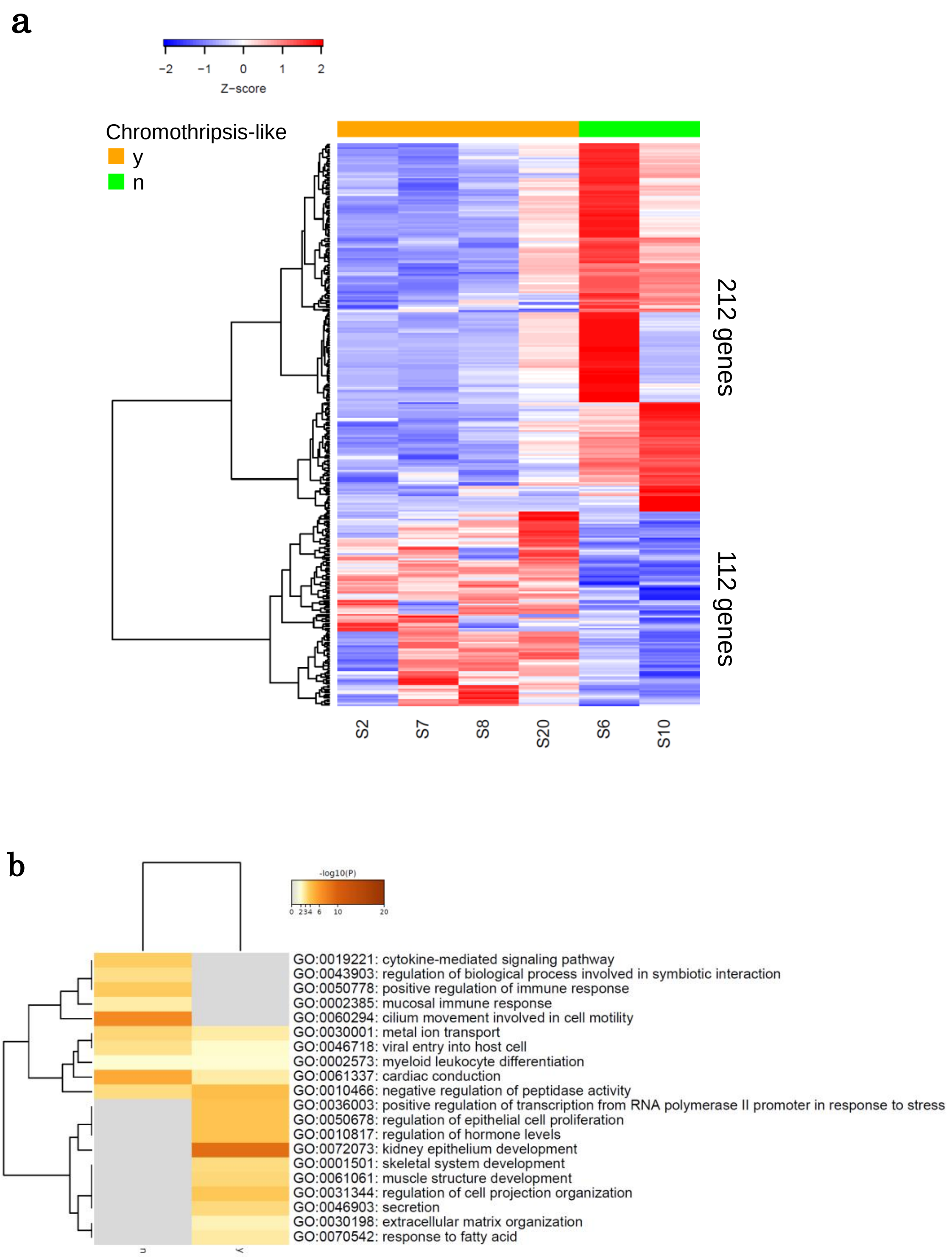

Supplementary Figure S16 Characterization of the chromothripsis event using gene expression patterns

(**a**) A heatmap of DEGs between the *EGFR* mutation-positive cases with and without the

chromothripsis-like event (p < 0.02; not adjusted, absolute value of log2 fold change > 1, DESeq2). The color keys are shown at the margin. (**b**) The result of gene enrichment analysis using the DEGs by Metascape (https://metascape.org/). The Gene Ontology Biological Process was used for this analysis. The multi-list enrichment analysis was conducted by Metascape under the default parameters, “Min Overlap = 3”, “P Value Cutoff = 0.01”, “Min Enrichment = 1.5.”

Note:
Genes associated with inflammatory and immune response pathways were highly expressed in chromothripsis-negative cases. The chromosomal missegregation and formation of micronuclei during the occurrence of chromothripsis, such as cGAS-STING pathway, would be censored by an immune surveillance system[16], resulting in immune response activation which plays an essential role in excluding tumor cells by the recruitment of immune cells. We suggested that in chromothripsis-positive cases, this immune response might not be activated and tumor cells with chromothripsis-like events would not be excluded. Immune escape ability in addition to the EGFR signaling activation would be crucial factors for the progression of chromothriptic lung cancers.

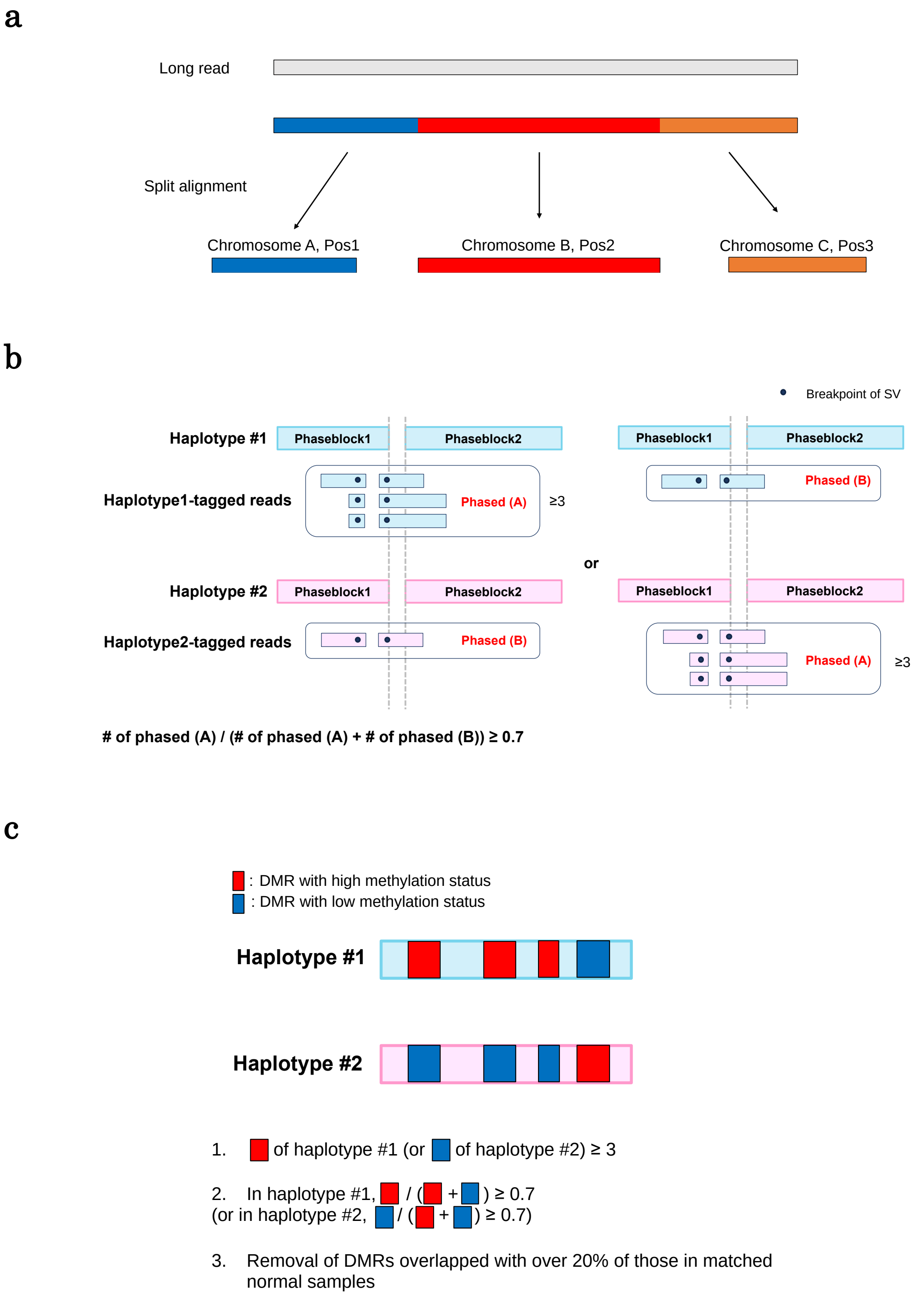

Supplementary Figure S17 Schemes for analysis of somatic SVs and DNA methylation with haplotype information

(a) Split alignment. (b) Scheme to identify somatic SVs and their haplotype information. (c) Scheme to detect tumor-specific “phase blocks with DMR bias.”

Supplementary Tables

Supplementary Table S1 The detailed statistics of normal genome phasing

Case	Total yields (Gb)	Depth	N50 read length	Number of blocks	N50 block length	Number of phased SNP	Coverage
S1	57	19	10,386	9,762	482,936	1,643,260	0.67
S2	41	14	15,046	10,680	494,768	1,875,692	0.78
S3	48	15	6,933	21,850	204,709	1,808,538	0.61
S5	35	11	11,404	14,268	327,653	1,807,273	0.70
S6	48	16	15,107	8,569	643,009	1,935,324	0.81
S7	46	15	16,773	8,952	593,620	1,927,627	0.80
S8	54	18	16,260	8,074	658,842	1,851,476	0.79
S9	84	28	18,993	4,645	1,165,957	1,874,658	0.85
S10	42	14	16,460	8,854	599,642	1,882,924	0.79
S11	59	19	14,319	9,416	547,783	1,846,203	0.74
S12	84	27	16,204	6,412	829,969	1,828,525	0.78
S13	38	12	15,199	13,507	374,835	1,863,718	0.74
S14	117	38	16,319	6,772	783,191	1,824,383	0.77
S15	55	18	16,479	7,045	737,663	1,892,553	0.77
S16	55	18	17,347	8,382	620,454	1,832,658	0.75
S17	59	19	21,430	5,347	1,007,078	1,844,046	0.80
S18	96	32	18,037	5,288	1,062,570	1,897,087	0.84
S19	82	26	16,713	5,820	893,531	1,817,031	0.79
S20	47	15	18,678	8,076	641,106	1,827,885	0.76
S21	57	19	16,812	8,816	634,053	1,880,725	0.79

Supplementary Table S2 The detailed statistics of tumor genome phasing

Case	Total yields (Gb)	Depth	N50 read length	Number of blocks	N50 block length	Number of phased SNP	Coverage
S1	99	33	19,213	5,056	997,143	1,645,747	0.72
S2	94	31	11,114	5,857	966,398	1,882,466	0.84
S3	77	25	14,479	6,035	870,215	1,820,266	0.79
S5	82	27	12,437	6,479	802,294	1,815,052	0.78
S6	76	25	19,617	4,988	1,137,963	1,938,893	0.85
S7	85	28	16,670	6,402	915,672	1,932,338	0.83
S8	100	33	16,894	5,356	1,096,149	1,853,440	0.84
S9	143	47	22,568	3,172	1,753,304	1,874,651	0.88
S10	85	28	19,756	4,542	1,236,789	1,889,156	0.85
S11	120	39	6,865	14,884	338,175	1,843,850	0.67
S12	125	40	15,804	4,806	1,122,594	1,829,220	0.81
S13	104	34	7,627	7,220	786,811	1,871,902	0.81
S14	118	38	11,385	9,746	546,448	1,821,384	0.73
S15	109	35	18,329	3,671	1,473,922	1,894,303	0.83
S16	104	34	5,957	17,044	284,887	1,825,459	0.67
S17	96	31	15,086	8,195	643,089	1,843,670	0.75
S18	62	20	23,446	4,710	1,140,556	1,896,280	0.85
S19	101	33	27,738	3,667	1,427,609	1,817,572	0.82
S20	90	29	26,048	4,594	1,165,711	1,830,392	0.81
S21	105	35	20,092	4,300	1,336,363	1,882,776	0.86

Supplementary Table S3 Discrepancies of phase information between tumor and normal genomes

Case	Phased pairs of variants assessed	Switch errors	Switch/flip	Switch error rate (%)
S1	1,631,157	10,308	2,834/3,737	0.63
S2	1,861,295	18,848	4,594/7,127	1.01
S3	1,784,067	19,506	5,264/7,121	1.09
S5	1,789,333	14,888	3,540/5,674	0.83
S6	1,922,820	17,224	4,222/6,501	0.90
S7	1,913,505	18,594	4,042/7,276	0.97
S8	1,839,557	15,054	3,282/5,886	0.82
S9	1,867,584	9,838	2,042/3,898	0.53
S10	1,870,956	18,890	3,670/7,610	1.01
S11	1,825,205	11,344	3,196/4,074	0.62
S12	1,819,608	9,735	2,509/3,613	0.54
S13	1,846,718	14,911	3,713/5,599	0.81
S14	1,808,712	14,167	2,971/5,598	0.78
S15	1,883,183	12,989	3,205/4,892	0.69
S16	1,803,320	17,050	4,038/6,506	0.95
S17	1,832,391	9,287	2,355/3,466	0.51
S18	1,887,727	10,682	2,558/4,062	0.57
S19	1,809,147	9,776	2,374/3,701	0.54
S20	1,816,841	13,579	3,433/5,073	0.75
S21	1,869,955	11,000	2,762/4,119	0.59
Avg.	1,834,154	13,884	3,330/5,277	0.76

Supplementary Table S4 Comparison of the obtained haplotype information with those of the THC dataset

Case	Tumor			Normal		
	Match	Mismatch	Percentage of Match	Match	Mismatch	Percentage of Match
S1	1,365,462	17,342	98.7	1,360,027	16,927	98.8
S2	1,567,809	20,355	98.7	1,557,487	22,155	98.6
S3	1,558,074	20,836	98.7	1,533,361	21,686	98.6
S5	1,552,772	20,637	98.7	1,540,017	20,574	98.7
S6	1,606,218	22,104	98.6	1,600,664	22,437	98.6
S7	1,606,485	21,242	98.7	1,599,732	22,651	98.6
S8	1,545,290	21,108	98.7	1,541,925	20,941	98.7
S9	1,568,709	20,717	98.7	1,568,171	19,960	98.7
S10	1,575,642	20,724	98.7	1,565,409	23,089	98.5
S11	1,567,632	18,404	98.8	1,574,070	19,101	98.8
S12	1,564,045	19,455	98.8	1,562,247	19,269	98.8
S13	1,561,038	20,264	98.7	1,549,253	21,128	98.7
S14	1,552,104	21,167	98.7	1,558,534	19,699	98.8
S15	1,612,985	20,408	98.8	1,608,934	20,170	98.8
S16	1,550,968	20,277	98.7	1,565,667	19,676	98.8
S17	1,576,700	18,913	98.8	1,579,145	19,670	98.8
S18	1,581,046	21,060	98.7	1,581,488	20,422	98.7
S19	1,554,412	20,431	98.7	1,552,441	19,923	98.7
S20	1,542,390	21,729	98.6	1,537,915	21,592	98.6
S21	1,565,650	20,533	98.7	1,561,108	19,808	98.7

Supplementary Table S5 The detailed statistics of the sequencing and phasing analysis results obtained from the PromethION Q20 platform

a. General statistics of sequencing

	HG002	S20-T	S2-T
Total yields (Gb)	33	35	27
Depth (×)	11	11	9
N50 read length (bp)	23,789	19,405	7,173
Sequencing identity (%)	96	96	97

b. General statistics of phasing analysis

	S20-T	S2-T
Number of blocks	10,262	31,352
N50 block length (bp)	504,275	154,802
Number of phased SNP	1,828,245	1,864,111
Coverage	0.74	0.67

Supplementary Table S6 The list of the mutation pairs of which the orders were resolved by both long reads and PyClone-VI analysis

Case	Mutation combination			PyClone cluster		PyClone CCF	
	Chromosome	Position					
		SNV-1	SNV-2	SNV-1	SNV-2	SNV-1	SNV-2
S3	chr14	29248491	29250697	C-1	C-2	1.000	0.515
S3	chr14	30164735	30165454	C-1	C-2	1.000	0.515
S3	chr14	38300557	38302364	C-2	C-1	0.515	1.000
S3	chr14	45714852	45716146	C-1	C-2	1.000	0.515
S3	chr14	81755694	81756411	C-2	C-1	0.515	1.000
S8	chr13	74953604	74954009	C-1	C-2	1.000	0.414
S8	chr21	30973248	30975958	C-2	C-1	0.414	1.000
S8	chr6	113659151	113659488	C-2	C-1	0.414	1.000
S10	chrX	67777621	67778632	C-1	C-2	0.996	0.842
S14	chr1	104101816	104102057	C-1	C-3	1.000	0.212
S14	chr1	240564634	240564695	C-3	C-1	0.212	1.000
S14	chr1	242572924	242574980	C-1	C-2	1.000	0.616
S14	chr10	44471613	44474204	C-3	C-1	0.212	1.000
S14	chr12	45015120	45015419	C-3	C-1	0.212	1.000
S14	chr14	83860489	83879898	C-1	C-3	1.000	0.212
S14	chr14	86944327	86947603	C-3	C-1	0.212	1.000
S14	chr15	87056101	87058063	C-1	C-3	1.000	0.212
S14	chr16	27074460	27076068	C-2	C-1	0.616	1.000
S14	chr16	51021956	51023979	C-1	C-2	1.000	0.616
S14	chr17	70231350	70231755	C-2	C-3	0.616	0.212
S14	chr18	51760057	51772708	C-3	C-1	0.212	1.000
S14	chr18	61319816	61329046	C-1	C-3	1.000	0.212
S14	chr2	11964708	11968051	C-3	C-1	0.212	1.000
S14	chr2	103956673	103967569	C-3	C-1	0.212	1.000
S14	chr2	124201108	124212512	C-3	C-1	0.212	1.000
S14	chr2	130746422	130748437	C-3	C-1	0.212	1.000
S14	chr2	142296607	142297528	C-1	C-3	1.000	0.212
S14	chr3	20351025	20355505	C-3	C-1	0.212	1.000

S14	chr3	96201280	96201381	C-1	C-3	1.000	0.212
S14	chr3	140978899	140979108	C-1	C-3	1.000	0.212
S14	chr4	14858480	14868244	C-2	C-1	0.616	1.000
S14	chr4	55037469	55039745	C-3	C-1	0.212	1.000
S14	chr5	13232566	13237413	C-1	C-3	1.000	0.212
S14	chr6	65955222	65962543	C-3	C-1	0.212	1.000
S14	chr7	44746152	44747381	C-1	C-3	1.000	0.212
S14	chr7	71764332	71765565	C-1	C-2	1.000	0.616
S14	chr7	119353585	119355921	C-1	C-2	1.000	0.616
S14	chr7	155870372	155871572	C-1	C-3	1.000	0.212
S14	chr8	9959151	9959505	C-1	C-3	1.000	0.212
S14	chr8	64432727	64436848	C-1	C-2	1.000	0.616
S14	chr8	87861941	87861943	C-3	C-1	0.212	1.000
S14	chr8	120423526	120424960	C-1	C-2	1.000	0.616
S14	chr9	22978513	22982749	C-3	C-2	0.212	0.616
S20	chr11	104148144	104153041	C-2	C-1	0.323	1.000

Note:
The order of the mutation occurrence from long read information was inconsistent with that from PyClone-VI in only one mutation pair (chr5:169655197 and chr5:169655661).

Supplementary Table S7 General statistics of scDNA-seq

	S8	S20
Number of cells	178	420
Number of reads	5,387,159,732	5,463,174,238
Median ploidy	2.87	1.74
Median estimated CNV resolution (in Mb)	0.40	0.43
Number of cells (after removing noisy cells)	**138**	**376**

Supplementary Table S8 Overlaps between A-to-I RNA editing sites and SNP sites

Case	Total phased SNPs	Phased SNPs (A>G)	Phased SNPs in RNA-seq data (A>G)
S1	1,645,747	258,123 (16%)	6,518 (0.40%)
S3	1,820,266	286,304 (16%)	9,333 (0.51%)
S6	1,938,893	303,024 (16%)	4,307 (0.22%)
S8	1,853,440	290,559 (16%)	10,114 (0.55%)
S10	1,889,156	286,304 (15%)	9,333 (0.49%)
S20	1,830,392	286,604 (16%)	5,451 (0.30%)

Supplementary Table S9 Haplotype-biased expressed genes associated with haplotype-specific regulatory events

	Number of haplotype-biased expressed genes				
	Breakdown of accompanied regulatory events				Total
	Both	Only regulatory mutation*	Only DMR**	Neither	
S1	0 (0%)	0 (%)	54 (5.4%)	954 (94.6%)	1008
S3	5 (0.4%)	110 (8.9%)	24 (2.0%)	1091 (88.7%)	1230
S6	0 (0%)	0 (0%)	2 (4.5%)	42 (95.5%)	44
S8	1 (0.1%)	9 (0.9%)	32 (3.3%)	933 (96.7%)	975
S10	2 (0.2%)	5 (0.6%)	59 (7.0%)	779 (92.2%)	845
S20	0 (0%)	1 (0.5%)	10 (4.9%)	193 (94.6%)	204
Average	1	21	30	665	718

*Haplotype-resolved mutations on promoter/enhancer regions were extracted as regulatory mutations.

**DMRs between haplotypes were assigned to the genes with ± 50 kb from TSS.

Supplementary Table S10 The candidates of transcription factor bindings in regulatory regions of the *CLN5* gene in case S10

a. Regulatory mutation (chr13:76990951, G>C, ±10 bp)

Motif	TF	WT						Mut						Annotation
		Start	End	Strand	Score	Relative score	P-value*	Start	End	Strand	Score	Relative score	P-value*	
MA1581.1	ZBTB6	5	17	+	9.13	0.86	3e-4	---	---	---	---	---	---	only WT
MA1655.1	ZNF341	9	20	+	8.72	0.85	2e-4	---	---	---	---	---	---	only WT
MA1649.1	ZBTB12	6	16	+	7.5	0.85	8e-4	---	---	---	---	---	---	only WT
MA1508.1	IKZF1	10	21	+	7.21	0.81	3e-4	10	21	+	6.89	0.81	5e-4	High in WT
MA0745.1	SNAI2	4	12	−	5.97	0.9	0.0015	4	12	−	9.46	0.95	6e-4	High in Mut
MA0106.2	TP53	---	---	---	---	---	---	6	20	+	5.96	0.85	2e-4	only Mut
MA1646.1	OSR2	---	---	---	---	---	---	8	19	+	8.25	0.84	7e-4	only Mut
MA0500.2	MYOG	---	---	---	---	---	---	8	19	+	6.19	0.83	7e-4	only Mut
MA1100.1	ASCL1	---	---	---	---	---	---	7	19	+	5.91	0.82	5e-4	only Mut
MA0522.3	TCF3	---	---	---	---	---	---	3	13	+	7.63	0.86	5e-4	only Mut
MA1100.2	ASCL1	---	---	---	---	---	---	9	18	−	7.72	0.9	8e-4	only Mut

*Empirical p-values for the scores based on the distribution of sampled scores were calculated using the R package TFBSTools[17]. No multiple comparison adjustments were performed.

b. DMR (chr13:76979577-76979862, 286 bp)

Motif	TF	Start	End	Strand	Score	Relative score
MA1513.1	KLF15	1	11	−	8.3	0.90
MA0489.1	JUN(var.2)	5	18	+	12.3	0.95
MA1101.2	BACH2	5	23	−	10.7	0.91
MA0477.2	FOSL1	8	20	+	12.4	0.93
MA0478.1	FOSL2	8	18	+	14.7	0.99
MA0490.1	JUNB	8	18	+	14.2	0.98
MA0490.2	JUNB	8	20	+	10.8	0.89
MA0491.2	JUND	8	20	+	11.5	0.91
MA1128.1	FOSL1::JUN	8	20	+	13.4	0.95
MA1134.1	FOS::JUNB	8	19	−	13.3	0.95
MA1141.1	FOS::JUND	8	20	−	13.9	0.96
MA0099.3	FOS::JUN	9	18	−	13.8	0.97
MA0476.1	FOS	9	19	+	13.5	0.98
MA0477.1	FOSL1	9	19	+	15.1	0.99
MA0491.1	JUND	9	19	+	14.1	0.98
MA0835.2	BATF3	9	19	+	10.2	0.89
MA0841.1	NFE2	9	19	+	10.9	0.94
MA1101.1	BACH2	9	22	+	9.6	0.84
MA1130.1	FOSL2::JUN	9	20	−	13.4	0.96
MA1132.1	JUN::JUNB	9	18	+	12.3	0.94
MA1135.1	FOSB::JUNB	9	18	+	14.0	0.97
MA1138.1	FOSL2::JUNB	9	18	+	13.9	0.97
MA1142.1	FOSL1::JUND	9	18	+	11.2	0.95
MA1144.1	FOSL2::JUND	9	18	+	14.1	0.97
MA1634.1	BATF	9	19	+	10.9	0.91
MA0501.1	MAF::NFE2	10	24	+	5.3	0.84
MA1656.1	ZNF449	29	42	+	11.7	0.88
MA0154.3	EBF1	37	50	−	4.7	0.85
MA1587.1	ZNF135	43	56	−	18.1	0.95
MA0499.2	MYOD1	53	65	−	10.3	0.88
MA0745.2	SNAI2	53	65	−	11.6	0.91

MA0830.2	TCF4	53	65	−	13.0	0.93
MA1631.1	ASCL1(var.2)	53	65	−	14.0	0.95
MA0103.3	ZEB1	54	64	−	12.2	0.97
MA0522.3	TCF3	54	64	−	12.8	0.97
MA0745.1	SNAI2	55	63	+	12.0	0.99
MA1558.1	SNAI1	55	64	+	13.1	0.99
MA0130.1	ZNF354C	60	65	−	9.9	1.00
MA1632.1	ATF2	67	79	+	9.0	0.86
MA0018.2	CREB1	70	77	+	11.0	0.95
MA0729.1	RARA	71	88	+	-2.2	0.83
MA1531.1	NR1D1	72	86	+	5.5	0.88
MA0106.3	TP53	87	104	−	4.3	0.82
MA0095.2	YY1	99	110	+	11.5	0.93
MA0080.4	SPI1	100	113	+	0.3	0.83
MA1103.2	FOXK2	113	123	−	9.3	0.88
MA0497.1	MEF2C	115	129	+	9.7	0.91
MA1125.1	ZNF384	117	128	+	16.9	1.00
MA0050.2	IRF1	120	140	−	11.3	0.86
MA0481.1	FOXP1	120	134	+	13.0	0.92
MA0517.1	STAT1::STAT2	131	145	−	5.8	0.83
MA1471.1	BARX2	135	146	+	11.9	0.96
MA0014.2	PAX5	139	157	+	10.3	0.88
MA0039.3	KLF4	146	156	−	12.9	0.95
MA0079.4	SP1	146	160	−	2.8	0.89
MA0747.1	SP8	146	157	−	9.0	0.89
MA1564.1	SP9	146	157	−	12.2	0.93
MA0599.1	KLF5	148	157	−	11.2	0.97
MA1515.1	KLF2	148	158	−	14.4	0.99
MA1516.1	KLF3	148	158	−	14.3	0.96
MA1517.1	KLF6	148	158	−	14.6	0.99
MA0799.1	RFX4	168	183	−	7.4	0.86
MA0516.1	SP2	214	228	−	12.4	0.93
MA1596.1	ZNF460	214	229	−	23.9	0.97
MA1653.1	ZNF148	215	226	−	10.2	0.86
MA0079.2	SP1	218	227	−	11.5	0.93
MA0079.3	SP1	218	228	−	12.8	0.97

MA0258.1	ESR2	225	242	−	8.9	0.86
MA0670.1	NFIA	246	255	+	11.7	0.98
MA0671.1	NFIX	246	254	+	10.3	0.99
MA1146.1	NR1H4::RXRA	246	260	−	10.5	0.84
MA0161.1	NFIC	248	253	−	9.8	1.00

Note: The p-value is smaller than 1e-4.

Supplementary Table S11 Comparison of clinical/pathological and genomic backgrounds information between the cases with/without SV-concentrated phased blocks

Category			SV-concentrated phased blocks		P-value† (Fisher's exact test, *Wilcoxon rank-sum test)
			y (6)	n (14)	
Age	Median [Range]		61 [50–71]	68.5 [38–85]	0.32*
Sex	Female		4	6	0.63
	Male		2	8	
Pathological Stage	IA1		1	0	0.18
	IA3		3	3	
	IB		0	3	
	IIB		2	2	
	IIIA		0	4	
	IIIB		0	2	
Subtype	Adenocarcinoma		5	10	1.0
	Others		1	4	
Necrosis	y		1	6	0.35
	n		5	8	
Inflammation	Mild		1	0	0.27
	Mild to moderate		1	1	
	Moderate		2	10	
	Mod to severe		1	1	
	Severe		1	2	
Tumor purity	Low		1	2	1.0
	Moderate		4	10	
	High		1	2	
Point mutations	Total	Median [Range]	6,651.5 [4,410–93,177]	6,580.5 [651–458,584]	0.97*
SVs	Total	Median [Range]	151 [64–2,490]	57.5 [14–603]	0.095*
	Translocation	Median	31.5	10.5	0.059*

	[Range]	[6–64]	[2–55]	
Inversion	Median	52	12	**0.026***
	[Range]	[22–2,261]	[1–445]	
Duplication	Median	18.5	2.5	**0.0016***
	[Range]	[4–36]	[0–14]	
Insertion	Median	12	9	0.51*
	[Range]	[5–52]	[0–149]	
Deletion	Median	35.5	17	0.30*
	[Range]	[12–77]	[2–134]	

†The p-values were calculated by Fisher's exact test or Wilcoxon rank-sum test (two-sided).

Supplementary References

1. Chen, S., Zhou, Y., Chen, Y. & Gu, J. Fastp: An ultra-fast all-in-one FASTQ preprocessor. *Bioinformatics* **34**, i884–i890 (2018).
2. Li, H. & Durbin, R. Fast and accurate short read alignment with Burrows-Wheeler transform. *Bioinformatics* **25**, 1754–1760 (2009).
3. Li, H. *et al.* The Sequence Alignment/Map format and SAMtools. *Bioinformatics* **25**, 2078–2079 (2009).
4. Li, H. A statistical framework for SNP calling, mutation discovery, association mapping and population genetical parameter estimation from sequencing data. *Bioinformatics* **27**, 2987–2993 (2011).
5. McKenna, A. *et al.* The genome analysis toolkit: A MapReduce framework for analyzing next-generation DNA sequencing data. *Genome Res.* **20**, 1297–1303 (2010).
6. Depristo, M. A. *et al.* A framework for variation discovery and genotyping using next-generation DNA sequencing data. *Nat. Genet.* **43**, 491–501 (2011).
7. Altshuler, D. M. *et al.* An integrated map of genetic variation from 1,092 human genomes. *Nature* **491**, 56–65 (2012).
8. Sherry, S. T. *et al.* dbSNP: the NCBI database of genetic variation. *Nucleic Acids Res.* **29**, 308–11 (2001).
9. Mills, R. E. *et al.* An initial map of insertion and deletion (INDEL) variation in the human genome. *Genome Res.* **16**, 1182–1190 (2006).
10. Belmont, J. W. *et al.* A haplotype map of the human genome. *Nature* **437**, 1299–1320 (2005).
11. Martin, M. *et al.* WhatsHap: fast and accurate read-based phasing. *bioRxiv* 085050 (2016). doi:10.1101/085050
12. Shiraishi, Y. *et al.* Precise characterization of somatic structural variations and mobile element insertions from paired long-read sequencing data with nanomonsv. *bioRxiv* 2020.07.22.214262 (2020). doi:10.1101/2020.07.22.214262
13. Simpson, J. T. *et al.* Detecting DNA cytosine methylation using nanopore sequencing. *Nat. Methods* **14**, 407–410 (2017).
14. Suzuki, A. *et al.* DBTSS/DBKERO for integrated analysis of transcriptional regulation. *Nucleic Acids Res.* **46**, D229–D238 (2018).
15. Cobaleda, Ć., Ṕerez-Caro, M., Vicente-Duẽ nas, C. & Śanchez-García, I. Function of the zinc-finger transcription factor SNAI2 in cancer and development. *Annu. Rev. Genet.* **41**, 41–61 (2007).
16. MacKenzie, K. J. *et al.* CGAS surveillance of micronuclei links genome instability to

innate immunity. *Nature* **548**, 461–465 (2017).

17. Tan, G. & Lenhard, B. TFBSTools: An R/bioconductor package for transcription factor binding site analysis. *Bioinformatics* **32**, 1555–1556 (2016).
